# A Genome-Scale Model of *Shewanella piezotolerans* Simulates Mechanisms of Metabolic Diversity and Energy Conservation

**DOI:** 10.1128/mSystems.00165-16

**Published:** 2017-03-28

**Authors:** Keith Dufault-Thompson, Huahua Jian, Ruixue Cheng, Jiefu Li, Fengping Wang, Ying Zhang

**Affiliations:** a Department of Cell and Molecular Biology, College of the Environment and Life Sciences, University of Rhode Island, Kingston, Rhode Island, USA; b Graduate School of Biological and Environmental Sciences, College of the Environment and Life Sciences, University of Rhode Island, Kingston, Rhode Island, USA; c State Key Laboratory of Microbial Metabolism, School of Life Sciences and Biotechnology, Shanghai Jiao Tong University, Shanghai, People’s Republic of China; Luxembourg Centre for Systems Biomedicine

**Keywords:** *Shewanella*, metabolic modeling

## Abstract

*Shewanella piezotolerans* strain WP3 belongs to the group 1 branch of the *Shewanella* genus and is a piezotolerant and psychrotolerant species isolated from the deep sea. In this study, a genome-scale model was constructed for WP3 using a combination of genome annotation, ortholog mapping, and physiological verification. The metabolic reconstruction contained 806 genes, 653 metabolites, and 922 reactions, including central metabolic functions that represented nonhomologous replacements between the group 1 and group 2 *Shewanella* species. Metabolic simulations with the WP3 model demonstrated consistency with existing knowledge about the physiology of the organism. A comparison of model simulations with experimental measurements verified the predicted growth profiles under increasing concentrations of carbon sources. The WP3 model was applied to study mechanisms of anaerobic respiration through investigating energy conservation, redox balancing, and the generation of proton motive force. Despite being an obligate respiratory organism, WP3 was predicted to use substrate-level phosphorylation as the primary source of energy conservation under anaerobic conditions, a trait previously identified in other *Shewanella* species. Further investigation of the ATP synthase activity revealed a positive correlation between the availability of reducing equivalents in the cell and the directionality of the ATP synthase reaction flux. Comparison of the WP3 model with an existing model of a group 2 species, *Shewanella oneidensis* MR-1, revealed that the WP3 model demonstrated greater flexibility in ATP production under the anaerobic conditions. Such flexibility could be advantageous to WP3 for its adaptation to fluctuating availability of organic carbon sources in the deep sea.

**IMPORTANCE** The well-studied nature of the metabolic diversity of *Shewanella* bacteria makes species from this genus a promising platform for investigating the evolution of carbon metabolism and energy conservation. The *Shewanella* phylogeny is diverged into two major branches, referred to as group 1 and group 2. While the genotype-phenotype connections of group 2 species have been extensively studied with metabolic modeling, a genome-scale model has been missing for the group 1 species. The metabolic reconstruction of *Shewanella piezotolerans* strain WP3 represented the first model for *Shewanella* group 1 and the first model among piezotolerant and psychrotolerant deep-sea bacteria. The model brought insights into the mechanisms of energy conservation in WP3 under anaerobic conditions and highlighted its metabolic flexibility in using diverse carbon sources. Overall, the model opens up new opportunities for investigating energy conservation and metabolic adaptation, and it provides a prototype for systems-level modeling of other deep-sea microorganisms.

## Abstract (precis)

The well-studied nature of the metabolic diversity of *Shewanella* bacteria makes species from this genus a promising platform for investigating the evolution of carbon metabolism and energy conservation. The *Shewanella* phylogeny is diverged into two major branches, referred to as group 1 and group 2. While the genotype-phenotype connections of group 2 species have been extensively studied with metabolic modeling, a genome-scale model has been missing for the group 1 species. The metabolic reconstruction of *Shewanella piezotolerans* strain WP3 represented the first model for *Shewanella* group 1 and the first model among piezotolerant and psychrotolerant deep-sea bacteria. The model brought insights into the mechanisms of energy conservation in WP3 under anaerobic conditions and highlighted its metabolic flexibility in using diverse carbon sources. Overall, the model opens up new opportunities for investigating energy conservation and metabolic adaptation, and it provides a prototype for systems-level modeling of other deep-sea microorganisms.

## INTRODUCTION

Members of the *Shewanella* genus are present in a wide range of environments, including fresh and salt waters, food products, sewage systems, and deep-sea sediments (1–3). The *Shewanella* genus is known to utilize diverse carbon sources and electron acceptors, leading to its broad adaptation to various environmental conditions (3–6). A 16S rRNA gene-based phylogenetic reconstruction has revealed two major groups in the *Shewanella* genus (7). Generally, group 1 includes species that are capable of producing eicosapentaenoic acid (EPA) and are piezotolerant and psychrotolerant, such as *Shewanella benthica* and *Shewanella violacea*, which have been isolated from the deep sea. Group 2 species are generally pressure sensitive and mesophilic and include *Shewanella oneidensis*, *Shewanella baltica*, and *Shewanella putreficans*, which have been isolated from a variety of environments, including fresh water lakes and spoiled meat products.

The ability of *Shewanella* species to utilize a broad range of electron acceptors makes this genus a target for studying metabolic energy conservation and anaerobic respiration. Several recent studies have focused on identifying the relative contributions of two distinct ATP-producing mechanisms (8–10), oxidative phosphorylation and substrate-level phosphorylation. Oxidative phosphorylation is typically associated with respiration, where the reduction of terminal electron acceptors is coupled to proton motive force (PMF) generation, and the PMF subsequently contributes to ATP synthesis via ATP synthase (ATPase). Substrate-level phosphorylation is associated with the production of ATP through direct transfer of a phosphoryl group to ADP through the action of enzymes like phosphotransacetylase (Pta) and acetate kinase (AckA). In *S. oneidensis* strain MR-1, substrate-level phosphorylation is the primary source of ATP during anaerobic growth, while ATPase has either minor contributions to ATP production or acts as an ATP-driven proton pump that generates PMF (8). This is surprising, given that *Shewanella* bacteria are obligated to utilize terminal electron acceptors when growing under anaerobic conditions. An understudied aspect of these features of metabolism is how ATP production, PMF generation, and redox reactions interact and jointly contribute to the utilization of metabolic pathways and energy conservation strategies in *Shewanella* bacteria.

*Shewanella piezotolerans* strain WP3, hereinafter referred to as WP3, has been isolated from western-Pacific sediment at a depth of 1,914 m. It is piezotolerant and psychrotolerant, reflecting its adaptations to the deep-sea environment (11). A 16S-based phylogeny suggests this organism belongs to group 1 of the *Shewanella* genus (12). The ability of WP3 to utilize diverse carbon sources and electron acceptors demonstrates a metabolic flexibility that is comparable with that of other *Shewanella* species (11, 13). The full genome of WP3 includes diverse *c*-type cytochrome genes, which support anaerobic respiration using various terminal electron acceptors, such as nitrate, iron, trimethylamine-*N*-oxide (TMAO), and dimethyl sulfoxide (DMSO) (13). WP3 is also known to produce EPA and alter its lipid content to contain more unsaturated and branched-chain fatty acids in low-temperature and high-pressure environments (14). These features enlist WP3 as a good representative of the group 1 *Shewanella* species.

Genome-scale models (GEMs) of metabolic networks have broad applications in phenotype prediction, evolutionary reconstruction, functional analysis, and metabolic engineering (15). By connecting a set of biochemical reactions with the enzymatic functions encoded in a genome, GEMs provide a framework for simulating the associations between genotypes and phenotypes (16–19). The reconstruction of genome-scale models can be challenging due to the complexity in managing diverse data sets and maintaining model consistency through iterative manual curations. These challenges have been addressed with the recent releases of tools and automated pipelines to facilitate the modeling process (20–23). GEMs are available for four group 2 *Shewanella* species, including *S. oneidensis* MR-1, *S. denitrificans*, *Shewanella* sp. strain MR-4, and *Shewanella* sp. strain W3-18-1 (24, 25), while currently no GEM is available for any group 1 species.

This study focuses on WP3 as a prototype for metabolic modeling among group 1 *Shewanella*. WP3 presents the conserved features of the group 1 *Shewanella* (e.g., piezotolerance, psychrotolerance, EPA production, etc.) and is a well-studied species in this group. Previous studies have provided detailed evidence related to the function and annotation of multiple key metabolic pathways in WP3, including nitrate utilization (26), DMSO respiration (27), iron reduction and biomineralization (28–30), and fatty acid synthesis (14). In addition to functional annotations, extensive data are available on the expression of key metabolic genes, connecting individual pathways with their functional roles under changing environmental conditions (31–36). These studies provide a broad knowledge base for constructing the WP3 GEM. Furthermore, WP3 has established protocols for genetic manipulations (37–40). The experimental accessibility of this organism would enable the verification of modeling outcomes and support future research on molecular adaptations through combined GEM simulation and experimental verification. Overall, WP3 serves as an ideal organism for modeling the metabolism of group 1 species in the *Shewanella* genus.

In this study, a GEM of WP3 was constructed and applied in simulating the carbon metabolism and energy conservation under both aerobic and anaerobic conditions. The model was verified based on the known physiology of the organism and new experimental data. Evolutionary analysis of the central metabolic genes revealed nonhomologous replacements between WP3 (and other group 1 species) and the group 2 *Shewanella* species. Comparing the WP3 model with the model of a group 2 representative, *S. oneidensis* strain MR-1 (hereinafter referred to as MR-1), revealed similarities and differences between the two organisms in their aerobic growth and anaerobic energy conservation.

## RESULTS

### Phylogenetic position of *Shewanella piezotolerans* WP3.

**FIG 1:**
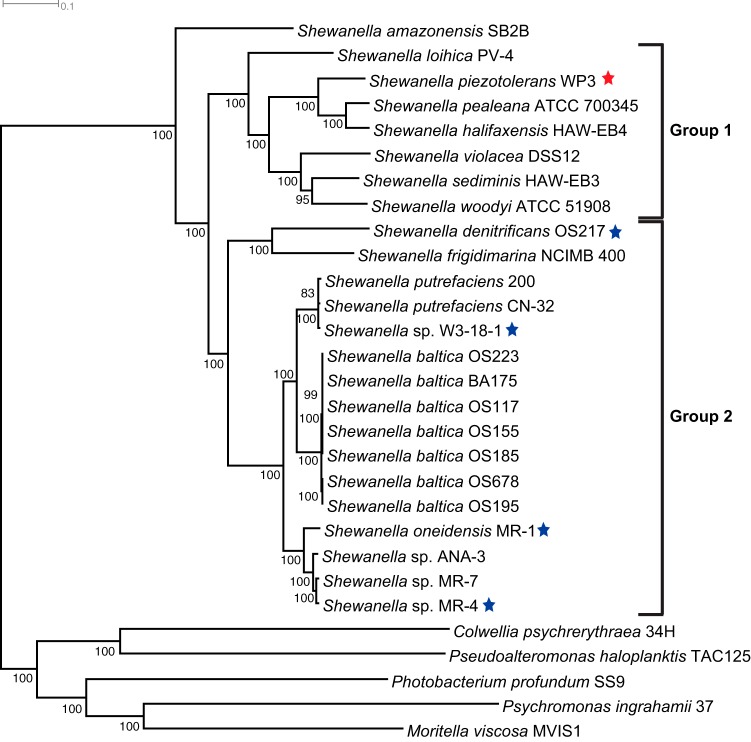
Phylogenetic reconstruction of the *Shewanella* genus based on the concatenated sequences of 661 conserved single-copy genes identified in the full genomes of *Shewanella* and five outgroup species. Support values based on 100 iterations of bootstrapping are indicated at the internal nodes. Only support values above 80 are shown. The four *Shewanella* species with available GEMs are marked with blue stars, and WP3 is marked with a red star.

The phylogenetic positioning of *S. piezotolerans* WP3 was confirmed following a phylogenomic analysis using the protein sequences of 661 conserved single-copy genes (CSCGs) in the full genomes of 24 *Shewanella* species and 5 closely related *Gammaproteobacteria* species that served as the outgroup to the *Shewanella* genus (see Materials and Methods). The phylogenomic reconstruction demonstrated the differentiation of the group 1 and group 2 *Shewanella* species into distinct evolutionary branches (Fig. 1) and concurred with a previously published 16S rRNA gene-based phylogeny (7). An exception to this concurrence was with the positioning of *Shewanella amazonensis*, where the 16S rRNA gene-based phylogeny located *S. amazonensis* in the group 2 taxa (7), while the genome-based phylogeny positioned *S. amazonensis* as one of the deepest-branching species among all of the *Shewanella* species analyzed. According to the genome-wide phylogeny, WP3 was located in the group 1 branch, with *Shewanella pealeana* and *Shewanella halifaxensis* as its closest neighbors. In the phylogenetic tree shown in Fig. 1, the four previously modeled *Shewanella* species are marked with blue stars to indicate their position in the phylogeny, while WP3 is marked with a red star.

### Genome-scale metabolic reconstruction of *Shewanella piezotolerans* WP3.

The complete metabolic reconstruction of WP3, GEM-iWP3, was released in a public Git repository at https://github.com/zhanglab/GEM-iWP3. It contained 806 genes, 653 metabolites, and 922 metabolic reactions. The reconstruction was achieved in three steps. First, gene-protein-reaction (GPR) associations were incorporated through mapping orthologous genes to the existing *Shewanella* reconstructions (24, 25) (see Materials and Methods). This identified 596 genes (619 reactions) that were conserved between WP3 and all four of the other *Shewanella* species modeled, as well as 130 genes (131 reactions) that were conserved between WP3 and some (but not all) of the four previously modeled species, leading to the inclusion of 726 genes associated with 750 reactions in the WP3 reconstruction.

**FIG 2:**
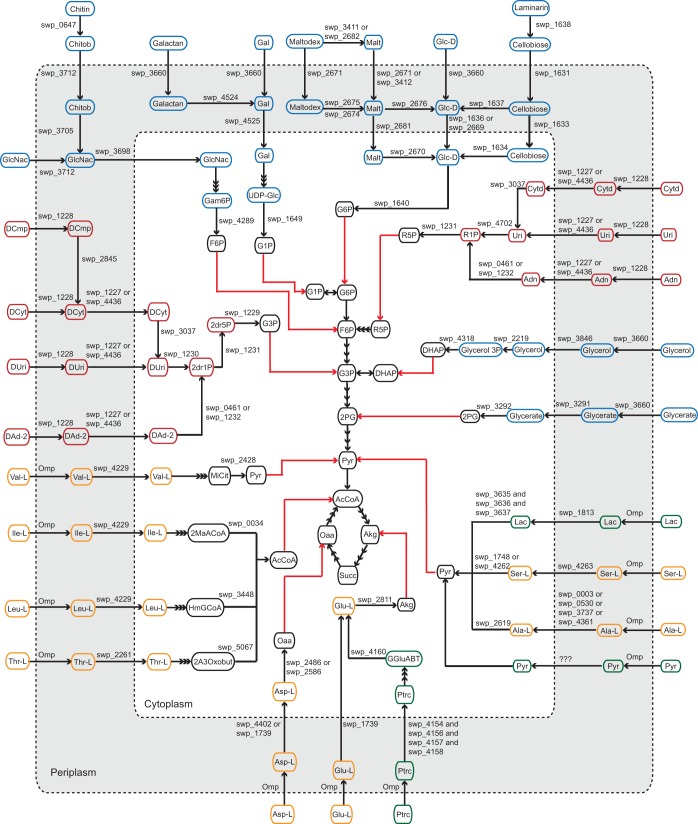
A schematic representation of the carbon utilization pathways for various carbohydrates and their derivatives (blue), amino acids (orange), nucleic acids (red), and small carbon molecules (green) as well as their links to the central carbon metabolism (red arrows). Metabolites are represented as ovals, and metabolic and transport reactions are represented as links between metabolites. Triple arrows linking two compounds indicate that multiple reactions are involved in the conversion of one compound to the other. Genes coding for the main metabolic steps of the carbon uptake pathways are indicated as labels above the links. Abbreviations: 2A3Oxobut, l-2-amino-3-oxobutanoate; 2dr1P, 2-deoxy-d-ribose 1-phosphate; 2dr5P, 2-deoxy-d-ribose 5-phosphate; 2MaACoA, 2-methylacetoacetyl-CoA; 2PG, d-glycerate 2-phosphate; AcCoA, acetyl-CoA; Adn, adenosine; Akg, 2-oxoglutarate; Ala-L, l-alanine; Asp-l, l-aspartate; Chitob, chitobiose; Cytd, cytidine; DAd-2, deoxyadenosine; DCmp, deoxycytidine monophosphate; DCyt, deoxycytidine; DHAP, dihydroxyacetone phosphate; DUri, deoxyuridine; F6P, d-fructose 6-phosphate; G1P, d-glucose 1-phosphate; G3P, glyceraldehyde 3-phosphate; G6P, d-glucose 6-phosphate; Gal, d-galactose; Gam6P, d-glucosamine 6-phosphate; GGluABT, gammaglutamyl-gamma-aminobutyrate; Glc-D, d-glucose; Glu-L, l-glutamate; HmGCoA, hydroxymethylglutaryl-CoA; Ile-L, l-isoleucine; Lac, lactate; Leu-L, l-leucine; Malt, maltose; Maltodex, maltodextrin; MiCit, methylisocitrate; Oaa, oxaloacetate; Ptrc, putrescine; Pyr, pyruvate; R1P, alpha-d-ribose 1-phosphate; R5P, alpha-d-ribose 5-phosphate; Ser-L, l-serine; Succ, succinate; Thr-L, l-threonine; UDP-Glc, UDP-glucose; Uri, uridine.; Val-L, l-valine.

Next, the WP3 metabolic reconstruction was expanded through manual curation of the WP3 genome using information from published literature (12, 14, 41), protein domain conservation, and evidence from the genomic and functional context of the metabolic genes (42). This expansion led to the inclusion of another 137 reactions associated with new gene annotations and the addition of a periplasmic compartment to account for cellular localizations of nutrient transporters and electron transport reactions. For example, the carbohydrate utilization pathways were annotated based on prior study of sugar catabolism in *Shewanella* (41) and further verified based on predictions of protein localization in the cell (Fig. 2) (43–45). The reduction of soluble electron acceptors, such as nitrate, nitrite, thiosulfate, and TMAO, was represented as periplasmic reactions, while the reduction of DMSO and oxidized metals, such as Fe(III), Mn(IV), uranium(VI), and chromium(VI), was represented as extracellular processes following existing knowledge of the cellular compartmentalization of the different electron transport processes in *Shewanella* species (46–55). Putative outer membrane transporters were identified and curated to identify what genes were responsible for nutrient exchange between the extracellular space and the periplasm. A number of nonspecific porins were identified, including distant homologs to the *Escherichia coli* OmpC and OmpF proteins (56), as well as a homolog to the OprF protein in *Pseudomonas aeruginosa* (57, 58). This analysis also identified functionally specific outer membrane proteins that were responsible for the uptake of carbohydrates (e.g., LamB and OprB), phosphate (OprP), cobalamin (BtuB), long-chain fatty acids (FadL), and nucleosides (Tsx) (41, 59–63).

The assembly of cell components in WP3 was represented with the addition of 8 synthesis reactions. The biomass equation was introduced to represent the composition of cell mass, including carbohydrates, proteins, RNA, DNA, lipids, vitamins, and cofactors (see Table S1 in the supplemental material). The stoichiometry of the biomass equation was normalized to reflect the millimole concentrations of individual components in 1 g of cell dry weight (gDW) (see Materials and Methods). The composition of macromolecules, such as lipids, proteins, DNA, and RNA, was represented using equations that defined the composition of basic building blocks, such as fatty acids, amino acids, and nucleotides. The stoichiometries of these biosynthesis equations were determined according to existing *Shewanella* reconstructions and experimental measurements performed on WP3. Specifically, the stoichiometry of the lipid biosynthesis equation (see Table S2) was calibrated based on experimentally measured concentrations of saturated, unsaturated, and branched-chain fatty acids in WP3 (14).

10.1128/mSystems.00165-16.5 TABLE S1  Stoichiometries of the biomass compounds involved in the biomass synthesis equation of the WP3 GEM. Download TABLE S1, PDF file, 0.02 MB. Copyright © 2017 Dufault-Thompson et al. 2017 Dufault-Thompson et al. This content is distributed under the terms of the Creative Commons Attribution 4.0 International license.

10.1128/mSystems.00165-16.6 TABLE S2  Stoichiometries of the fatty acid components in the lipid biosynthesis equation of the WP3 GEM. The stoichiometries of unsaturated, saturated, and branched-chain fatty acids were calibrated based on experimental measurements of the WP3 fatty acid composition at 20°C and 0.1 MPa (14). Download TABLE S2, PDF file, 0.01 MB. Copyright © 2017 Dufault-Thompson et al. 2017 Dufault-Thompson et al. This content is distributed under the terms of the Creative Commons Attribution 4.0 International license.

The WP3 metabolic reconstruction also included three reactions for the diffusion of O_2_, CO_2_, and urea across the cell membrane and 24 gap-filling reactions for unblocking the production of biomass components. These gap-filling reactions reflected knowledge gaps in the synthesis of biomass compounds, where the metabolic mechanisms were either unknown or not yet associated with any annotated genes in the WP3 genome. These gap-filling reactions included dihydroneopterin mono- and triphosphate dephosphorylases, which were involved in the synthesis of the cofactor tetrahydrobiopterin, as well as glycolaldehyde dehydrogenase and 5,10-methylenetetrahydrofolate reductase, which were involved in folate metabolism. Three gap-filling reactions were compound sinks that allowed for the removal of metabolic side products whose metabolic pathways are currently unknown and that are not involved in other reactions in the metabolic network. These included sinks for the compound *S*-adenosyl-4-methylthio-2-oxobutanoate, a side product in the synthesis of biotin.

Finally, 109 exchange reactions were defined to represent the exchange of nutrients and metabolic products in the simulated environment (see Table S3 in the supplemental material). These included reactions for the uptake of carbon sources, electron acceptors, trace metals, and vitamin precursors, as well as the diffusion of metabolic by-products. These exchange reactions were set to represent the basal constraints specified in Table S3 and were subsequently modified during metabolic simulations to represent different environmental conditions (see Materials and Methods).

10.1128/mSystems.00165-16.7 TABLE S3  Basal constraints for metabolic simulations performed in the WP3 and MR-1 models (see Materials and Methods). “Compound ID/Name” lists the identifiers/names of extracellular compounds with defined exchange reactions, which were used to simulate the availability of nutrients and the removal of metabolic by-products. The compound identifiers are shown for both the WP3 and MR-1 models. “Lower/Upper Bound” lists basal constraints for the lower and upper bounds of exchange reaction fluxes. Negative lower bounds indicate compounds provided as nutrient sources to the model, and a lower bound of zero indicates a compound that could only be released as a metabolic by-product but not acquired from the environment. “Type” lists the classification of the exchange compounds. “Growth supporting in WP3” lists the growth-supporting carbon sources, and terminal electron acceptors are marked as TRUE in this column. Download TABLE S3, PDF file, 0.03 MB. Copyright © 2017 Dufault-Thompson et al. 2017 Dufault-Thompson et al. This content is distributed under the terms of the Creative Commons Attribution 4.0 International license.

### Evolution of central metabolic genes.

During manual curation of the WP3 metabolic reconstruction, genes for carrying out central metabolic functions that were nonhomologous between WP3 and the previously modeled *Shewanella* species were identified. These included acetylornithine deacetylase (*argE*), which is essential for the biosynthesis of arginine, and glucosamine-6-phosphate deaminase (*nagB*), which is essential for utilizing *N*-acetyl-d-glucosamine (GlcNac). Both genes were experimentally identified in MR-1 and were found to be nonhomologous to the canonical genes in *E. coli* (64, 65). A broader comparison of the group 1 and group 2 *Shewanella* species suggested that they were conserved within each group but had diverged between the two groups (see Fig. S1 in the supplemental material). Exceptions were found for the *argE* gene in *S. amazonensis*, *Shewanella loihica*, and *Shewanella frigidimarina*, where the deep-branching *S. amazonensis* and *S. loihica* carried both nonhomologous copies of *argE* and the group 2 species *S. frigidimarina* carried a single *argE* of the group 1 type. The genomic contexts of *argE* and *nagB* were well conserved among the group 1 species, while they were variable among the group 2 species. Consistent with the observed variability of the genomic contexts, genetic elements were found in proximity to *argE* and *nagB* in MR-1, as well as an *argE* in *S. loihica* that is homologous to the group 2 type. The group 2 genes had diverse origins, with the *argE* being homologous to genes in *Klebsiella* species and a limited subset of host-associated *Enterobacteriaceae* and the *nagB* homologous to genes in the deep-branching bacteria and archaea (65). In contrast, the group 1 genes were conserved with those of genera evolutionarily related to *Shewanella*, such as *Marinomonas*, *Colwellia*, and *Pseudoalteromonas*. Taken together, the central metabolic genes *argE* and *nagB* evolved from distinct origins among *Shewanella* groups 1 and 2. The WP3 genome carried the gene copies that were conserved in the group 1 species.

10.1128/mSystems.00165-16.1 FIG S1  Phylogenetic trees of ArgE and NagB proteins encoded in the genomes of group 1 (A and C) and group 2 (B and D) *Shewanella* species. Support values based on 100 iterations of bootstrapping are indicated at the internal nodes. Only support values above 80 are shown. The group 1 and group 2 copies of the corresponding proteins had no detectable homology, indicating nonhomologous replacements of the ArgE and NagB functions in the two groups of *Shewanella* species. Download FIG S1, PDF file, 0.4 MB. Copyright © 2017 Dufault-Thompson et al. 2017 Dufault-Thompson et al. This content is distributed under the terms of the Creative Commons Attribution 4.0 International license.

### Metabolic simulations match experimental growth measurements.

Simulations of biomass production with the WP3 metabolic model were consistent with the known physiology of this organism. This included utilizing glucose, lactate, maltose, and GlcNac as carbon sources and using Fe(III), nitrate, nitrite, thiosulfate, TMAO, and DMSO as terminal electron acceptors for anaerobic respiration (13). From the metabolic simulations, 53 sole carbon sources supported biomass production of the WP3 model under aerobic conditions, including various carbohydrates, amino acids, nucleotides, and fatty acids (Fig. 2; see also Table S3 in the supplemental material).

To quantitatively evaluate the prediction of biomass concentrations by the WP3 metabolic model, batch cultures were set up using a minimal medium developed in this study to experimentally measure the growth of WP3 with sole carbon sources (see Text S1 in the supplemental material). The sole carbon sources examined in this study were pyruvate, glucose, maltose, and an amino sugar (GlcNac), and the experiments were carried out under aerobic conditions using oxygen as the sole terminal electron acceptor. The concentrations of carbon sources ranged between 2 mM and 40 mM in the experimental medium. Cell growth was measured in three independent replicates and converted to biomass concentrations (see Materials and Methods). Metabolic simulations were performed with the WP3 model to predict the biomass fluxes under the conditions defined by the experimental medium. This was achieved by modifying the flux bounds of the exchange reactions in the model. The lower bounds of exchange reactions for carbon, nitrogen, sulfur, and phosphorus sources were specifically calibrated to reflect their concentrations in the minimal medium (see Table 1), the exchange of oxygen was unlimited to simulate aerobic respiration, and the lower and upper bounds of other exchange reactions were assigned based on default settings in the basal constraints (see Materials and Methods; see also Table S3 in the supplemental material).

10.1128/mSystems.00165-16.9 TEXT S1  Components of the LMO-812 minimal medium used for the experimental culture of *Shewanella piezotolerans* WP3. Medium components were adapted from a previously described defined marine medium (F. Widdel, p. 102–104, in *HERMES Handbook of Methods for Microbial Ecology*, 2005, accessible at https://epic.awi.de/29169/1/HER2005l.pdf). Download TEXT S1, PDF file, 0.1 MB. Copyright © 2017 Dufault-Thompson et al. 2017 Dufault-Thompson et al. This content is distributed under the terms of the Creative Commons Attribution 4.0 International license.

**TABLE 1:** Exchange reaction constraints representing the concentrations of carbon, nitrogen, sulfur, and phosphorus sources in the minimal medium of WP3 batch cultures^a^

Nutrient	Source	Concn	Flux bound of exchange reaction	
			Lower	Upper
Carbon	Glucose, maltose, GlcNac, or pyruvate	2 mM	−2.00	1,000.00
		5 mM	−5.00	1,000.00
		10 mM	−10.00	1,000.00
		20 mM	−20.00	1,000.00
		40 mM	−40.00	1,000.00
Sulfur	SO_4_	9.8 mM	−9.80	1,000.00
Phosphorus	PO_4_	0.7 mM	−0.70	1,000.00
Nitrogen	NH_4_	5.6 mM	−5.60	1,000.00

a All other exchange reactions in the WP3 model were defined with settings in the basal constraints. The compounds pyruvate, glucose, maltose, and GlcNac were used as sole carbon sources. The lower and upper bounds of exchange reaction fluxes are shown; negative values indicate that uptake of the nutrient was permitted. The concentrations of the sole carbon sources varied from 2 mM to 40 mM; the concentrations of the sulfur, phosphorus, and nitrogen sources were set according to their concentration in the experimental medium.

**FIG 3:**
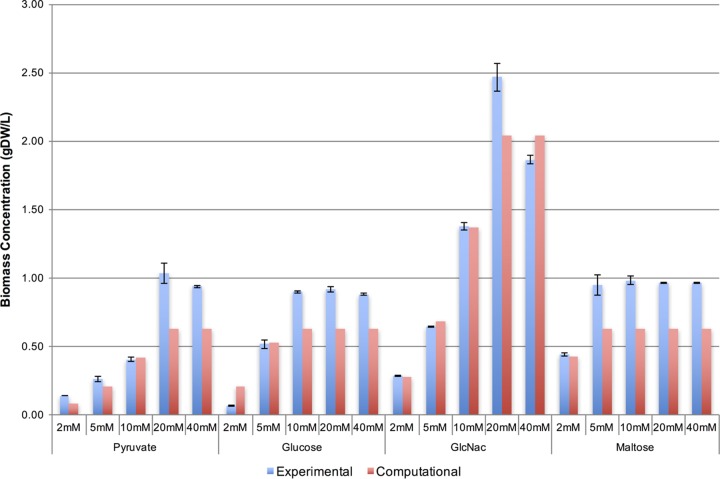
Comparison of experimentally measured and computationally simulated biomass production levels of WP3 grown with different carbon sources. Error bars represent the standard deviations of the experimentally measured biomass concentrations (gDW/liter) from three independent replicates.

The biomass fluxes predicted by the model demonstrated overall consistency with experimentally measured biomass concentrations at the stationary phase (Fig. 3). The quantitative values deviated slightly from the experimental measurements at relatively low (i.e., 2 mM of pyruvate or glucose) or high carbon source concentrations. Experimental measurements showed that the biomass production stopped increasing when the concentration of sole carbon sources increased beyond 60 mM in the count of carbon elements (i.e., 20 mM, 10 mM, or 5 mM of pyruvate [3 carbons], glucose [6 carbons], or maltose [12 carbons], respectively). This trend was also seen in the WP3 model simulations. Furthermore, under high concentrations of pyruvate, glucose, and maltose, metabolic simulations identified NH_4_^+^ as the limiting factor of biomass production. This was because the uptake of these carbon sources was limited by the uptake bound of the NH_4_^+^ exchange flux, which corresponded to its concentration in the experimental medium. Allowing for higher uptake of NH_4_^+^ by the model led to higher biomass production and higher uptake of these carbon sources. In contrast, the model was not limited by the availability of NH_4_^+^ when an amino sugar, GlcNac, was used as a sole carbon source. This was because each molecule of GlcNac produced one molecule of NH_4_^+^ during its utilization, providing additional nitrogen that could be used during growth. As a result, higher biomass was observed with GlcNac as a carbon source, and this trend was seen in both the experimental measurements and the model simulations (Fig. 3).

The aerobic growth of WP3 was also compared with that of MR-1 (model iMR1_799 [25]), based on simulations of growth on 28 sole carbon sources that have been experimentally confirmed to support growth in either WP3 (13) or MR-1 (24, 25) (see Fig. S2 in the supplemental material). The MR-1 model was able to utilize almost all the carbon sources examined, except for maltose. The WP3 model, in contrast, was viable in maltose but was not able to utilize six carbon sources, including the amino acids asparagine and glutamine, the nucleic acids inosine and thymidine, and the small molecules ethanol and 2-oxoglutarate. Simulations of biomass production using the two models revealed that WP3 had slightly higher biomass yields than MR-1 on most of the growth-supporting carbon sources, including carbohydrates, small carbon molecules, and amino acids, while MR-1 had a slightly higher biomass yield when malate, adenosine, or deoxyadenosine was used as the sole carbon source.

10.1128/mSystems.00165-16.2 FIG S2  Biomass yields for WP3 and MR-1 simulated across 28 carbon sources under aerobic conditions. Download FIG S2, PDF file, 0.1 MB. Copyright © 2017 Dufault-Thompson et al. 2017 Dufault-Thompson et al. This content is distributed under the terms of the Creative Commons Attribution 4.0 International license.

### Metabolic energy conservation of WP3.

**FIG 4:**
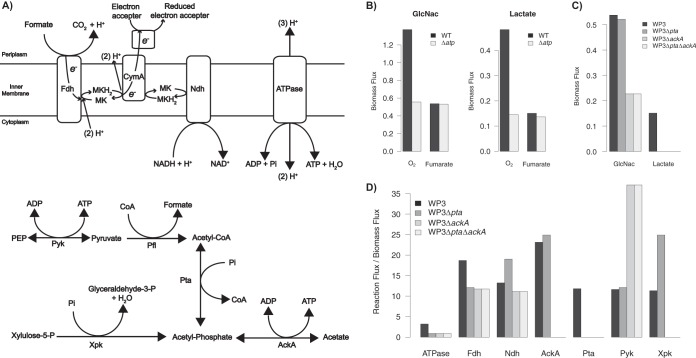
(A) A schematic representation of key reactions involved in the production of ATP and PMF in WP3. (B) Comparison of biomass fluxes in the wild-type and *Δatp* mutant models of WP3 with GlcNac or lactate as a sole carbon source under aerobic and anaerobic conditions. (C) Biomass fluxes from anaerobic growth simulations of the WP3 wild-type model and the *ΔackA*, *Δpta*, and *Δpta ΔackA* mutant models using GlcNac or lactate as a sole carbon source and fumarate as a sole electron acceptor. (D) Internal reaction fluxes of the WP3 and mutant models from the simulations whose results are shown in panel C, using GlcNac as a sole carbon source. MK, menaquinone; CymA, tetraheme *c*-type cytochrome; ATPase, ATP synthase; Fdh, formate dehydrogenase; Ndh, NADH dehydrogenase; AckA, acetate kinase; Pra, phosphotransacetylase; Pyk, pyruvate kinase; Xpk, xylulose-5-phosphate phosphoketolase.

The relative roles of oxidative and substrate-level phosphorylation were examined by simulating mutant models with reactions from each of the two pathways blocked (Fig. 4 and Materials and Methods). For measuring the role of oxidative phosphorylation, the levels of biomass production of the wild-type (WT) model and an ATP synthase deletion mutant (*Δatp* mutant) were simulated using flux balance analysis (FBA) under both aerobic (O_2_) and anaerobic (fumarate) conditions using either GlcNac or lactate as the sole carbon source. Under aerobic conditions, the *Δatp* mutant produced less than half of the WT biomass, indicating that oxidative phosphorylation played an important role in the aerobic growth of WP3. Under anaerobic conditions, however, the biomass production levels were comparable between the WT and *Δatp* models, demonstrating that oxidative phosphorylation had only a minor role in supporting anaerobic growth (Fig. 4B).

For measuring the role of substrate-level phosphorylation, FBA was performed with the WP3 WT model and three mutant models that represent the single deletion of the phosphotransacetylase (*Δpta* mutant) or the acetate kinase (*ΔackA* mutant) gene or the double deletion of both genes (*Δpta ΔackA* mutant). When lactate was used as a sole carbon source, the WT model was able to produce nonzero biomass flux, while the *Δpta*, *ΔackA*, and *Δpta ΔackA* models had a maximum biomass flux of zero, indicating that these mutants are not viable in the lactate medium. When GlcNac was used as a sole carbon source, both the WT and mutant models were viable in the anaerobic medium. Compared to the WT, the *Δpta* mutant had a slight decrease in biomass production (97% of the WT flux), and the *ΔackA* and *Δpta ΔackA* mutants resulted in greater reductions of the biomass, to less than 50% of the WT level (Fig. 4C). The decrease or inhibition of biomass production in the *ΔackA* and *Δpta ΔackA* mutants indicated an important role of substrate-level phosphorylation in supporting anaerobic growth of WP3.

Additional examination of the internal fluxes obtained from FBA revealed changes in ATP production, PMF generation, and redox functions in the WT and mutant models (Fig. 4D). In the WT model, substrate-level phosphorylation mediated by AckA was used for ATP production, while oxidative phosphorylation via ATP synthase (ATPase) played a minor role in this process. The activity of formate dehydrogenase (Fdh) was coupled with terminal electron acceptor reduction to generate PMF, and NADH dehydrogenase (Ndh) was used for reducing the quinone pool. In the *Δpta* model, reaction fluxes were redirected to xylulose-5-phosphate phosphoketolase (Xpk) from the pentose phosphate pathway so that substrate-level phosphorylation through AckA was maintained. This redirection resulted in reduced Fdh flux, potentially due to decreases in formate production, and increased Ndh flux, potentially for maintaining the redox activities in the electron transport chain. In the *ΔackA* and *Δpta ΔackA* models, more significant shifts were observed in the distribution of metabolic fluxes. The inhibition of AckA led to blockage of the upstream fluxes through Pta and Xpk and increased flux through pyruvate kinase (Pyk) to partially compensate for the loss of AckA-mediated ATP production.

The variability of the internal fluxes was further examined using flux variability analysis (FVA) with biomass production constrained to its maximum under each simulation condition (see Table S4 in the supplemental material). This revealed consistent flux values for all of the above-mentioned reactions in the WT and *Δpta* models and for the ATPase, AckA, Pta, and Xpk reactions in the *ΔackA* and *Δpta ΔackA* models. However, the Pyk, Fdh, and Ndh reactions had various flux values in the *ΔackA* and *Δpta ΔackA* mutants, indicating that these mutants had alternative strategies for balancing the ATP production, PMF generation, and redox activities in the cell.

10.1128/mSystems.00165-16.8 TABLE S4  Maximum and minimum flux values obtained from flux variability analysis (FVA), corresponding to the simulation conditions of the experiments whose results are shown in Fig. 4D. FVA was performed in the WP3 wild-type model and the *Δpta*, *ΔackA*, and *Δpta ΔackA* mutant models with biomass production set to its maximum (see Materials and Methods). Numbers in this table indicate raw values of the minimum and maximum fluxes before they were normalized by the biomass flux. Download TABLE S4, PDF file, 0.02 MB. Copyright © 2017 Dufault-Thompson et al. 2017 Dufault-Thompson et al. This content is distributed under the terms of the Creative Commons Attribution 4.0 International license.

### ATPase activity and anaerobic growth of *Shewanella*.

One surprising feature of the *Shewanella* anaerobic growth was the lack of oxidative phosphorylation via ATPase despite the obligate requirement for respiration through terminal electron acceptors (8). To further investigate how the ATPase activity (i.e., in either the ATP production or PMF generation direction) was related to the redox balancing of *Shewanella* during anaerobic respiration, the NAD^+^/NADH homeostasis was modeled with a robustness analysis to simulate the connections between redox state (as measured by the differences in NAD^+^ and NADH concentrations) and the activity of ATPase in both the WP3 and the MR-1 model (see Materials and Methods). The simulation demonstrated a positive correlation between the availability of reducing equivalents and the flux of the ATPase reaction for both WP3 and MR-1 (see Fig. S3 and S4 in the supplemental material). This indicated that when the system was provided with more reducing equivalents, the ATPase flux would increase and activity would be shifted toward the ATP-producing direction. In contrast, when the system had less reducing equivalents, the ATPase flux would decrease and activity would be flipped to the proton-pumping and PMF-generating direction.

10.1128/mSystems.00165-16.3 FIG S3  Linear models for the prediction of NAD^+^/NADH homeostasis in the WP3 model (see Materials and Methods). Fluxes of the ATPase reaction (black dots) were plotted based on a robustness simulation across varied fluxes of the EQ1 reaction. Linear models (red lines) were fitted to the observed correlations between EQ1 and ATPase fluxes and used to calculate the differences in NAD^+^ and NADH concentrations where the ATPase flux approached zero. Download FIG S3, PDF file, 0.04 MB. Copyright © 2017 Dufault-Thompson et al. 2017 Dufault-Thompson et al. This content is distributed under the terms of the Creative Commons Attribution 4.0 International license.

10.1128/mSystems.00165-16.4 FIG S4  Linear models for the prediction of NAD^+^/NADH homeostasis in the MR-1 model (see Materials and Methods). Fluxes of the ATPase reaction (black dots) were plotted based on a robustness simulation across varied fluxes of the EQ1 reaction. Linear models (red lines) were fitted to the observed correlations between EQ1 and ATPase fluxes and used to calculate the differences in NAD^+^ and NADH concentrations where the ATPase flux approached zero. Download FIG S4, PDF file, 0.04 MB. Copyright © 2017 Dufault-Thompson et al. 2017 Dufault-Thompson et al. This content is distributed under the terms of the Creative Commons Attribution 4.0 International license.

**FIG 5:**
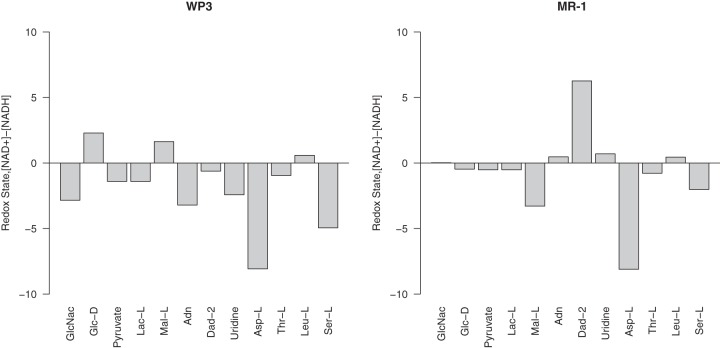
Comparison of NAD^+^/NADH homeostasis between WP3 (A) and MR-1 (B). The differences in NAD^+^ and NADH concentrations were calculated from simulations under anaerobic conditions with fumarate as the terminal electron acceptor for each of the carbon sources shown (*x* axis).

The comparison of redox states in the WP3 and MR-1 models when the ATPase reaction flux approached zero revealed metabolic differences between these two organisms across diverse carbon sources when using fumarate as the sole electron acceptor (Fig. 5). The WP3 model produced excess reducing equivalents with a wide range of carbon sources, including amino sugars, small carbon compounds, amino acids, and nucleotides (Fig. 5A). Considering the positive correlation of the redox state and the ATPase flux (see Fig. S3 and S4 in the supplemental material), the excess reducing equivalents in WP3 could potentially enable the production of additional ATP via ATPase. The MR-1 model, in contrast, produced excess reducing equivalents only when specific carbon sources were provided, such as malate, aspartate, and serine. Thus, the ATPase could have little contribution to the ATP production but may instead be used for PMF generation in MR-1. Overall, the two representatives of group 1 and group 2 *Shewanella* species, WP3 and MR-1, demonstrated complex interactions of ATP generation, PMF generation, and redox-balancing processes under anaerobic growth conditions. The WP3 model displayed higher capacity than MR-1 in producing excess reducing equivalents with most of the examined carbon sources. This may provide additional advantages to WP3 in its natural environment by enabling additional ATP production when using a diverse range of carbon sources.

## DISCUSSION

In this study, a genome-scale model was constructed for WP3, a piezotolerant and psychrotolerant representative of the group 1 *Shewanella* species (Fig. 1). Extensive annotations of the WP3 genome were incorporated into the metabolic reconstruction, and the carbon utilization reactions were curated based on the current literature available (Fig. 2). A periplasmic compartment was introduced to the WP3 reconstruction to account for the cellular localization of carbon utilization and electron transport reactions. This represented a new component not previously included by other metabolic reconstructions of *Shewanella* species.

Evolutionary analysis of central metabolic genes in WP3 revealed instances of nonhomologous replacements among the group 1 and group 2 *Shewanella* species. The *argE* and *nagB* in WP3 and other group 1 species were conserved within bacterial species closely related to *Shewanella*. Hence, they could represent the ancestral genes conserved during early differentiation of the *Shewanella* genus. The group 2 copies of these genes were adjacent to mobile genetic elements, suggesting a possible acquisition of these genes through horizontal gene transfer. Furthermore, the conservation of these acquired genes across group 2 species and their presence in a few group 1 species suggested they could have been introduced to the genome during early differentiation of the group 2 *Shewanella*.

The WP3 model represented the known physiology of this organism, including its growth with a wide variety of carbon sources and electron acceptors. A comparison of biomass production from model simulations and experimental measurements revealed that the WP3 model represented growth trends consistent with what was observed in experimental cultures using the sole carbon sources pyruvate, glucose, GlcNac, and maltose (Fig. 3). The slight deviations from the experimental results under low or excess carbon concentrations could be attributed to the differential regulation of gene expressions but were beyond the scope of this study. Additional confidence in the WP3 model was established when it was applied for the prediction of growth-limiting nutrients. The prediction of NH_4_^+^ as the limiting nutrient when an excess carbon source was provided was corroborated by the fact that the amide molecule, GlcNac, was able to overcome this growth limit by serving as both a carbon and a nitrogen source.

A comparison of the WP3 model with an existing model of a group 2 representative, *S. oneidensis* MR-1, revealed similarities and differences in carbon utilization and energy conservation of these two organisms. While MR-1 lacked enzymes for utilizing maltose, WP3 lacked identified transporters that are required for utilizing six carbon sources, including the amino acids asparagine and glutamine, the nucleic acids inosine and thymidine, and the small molecules ethanol and 2-oxoglutarate (see Fig. S2 in the supplemental material). The anaerobic energy conservation strategies of WP3 were explored by simulating the deletion of genes responsible for oxidative (*Δatp*) and substrate-level (*ΔackA*, *Δpta*, and *Δpta ΔackA*) phosphorylation (Fig. 4). Using GlcNac and lactate as sole carbon sources and fumarate as the sole electron acceptor, the biomass production and reaction flux distributions of the WP3 wild-type and mutant models revealed that substrate-level phosphorylation was the primary source of anaerobic energy conservation, a trait that has been noted in MR-1 (8, 10). This indicates that the primary usage of substrate-level phosphorylation could be a conserved feature in the anaerobic respiration of both group 1 and group 2 species and suggests that this feature could have evolved during the early differentiation of *Shewanella*.

Internal redox balancing has been shown to play a critical role in the ability of other organisms to utilize nutrients and is a major driver of changes in metabolic strategy (66–68). Simulation of the NAD^+^/NADH homeostasis and its connections to the ATPase activity in WP3 and MR-1 provided insights into the complex interactions of ATP production, PMF generation, and redox-balancing processes in *Shewanella* bacteria. While both the WP3 and MR-1 models presented a positive correlation between the availability of reducing equivalents and the reaction flux of ATPase (see Fig. S3 and S4 in the supplemental material), the two models demonstrated distinct redox states when different carbon sources were utilized (Fig. 5). The production of excess reducing equivalents was supported by a wide range of carbon sources in WP3 but was restricted to only a few carbon sources in MR-1. This suggested a capacity for WP3 to produce additional ATP via the ATPase activity and could potentially have enabled the adaptation of WP3 to the fluctuating availability of carbon sources in the deep sea by maintaining ATP production when different carbon sources became available.

Overall, the WP3 model represents the first genome-scale model of the group 1 *Shewanella* species and the first model of a piezotolerant and psychrotolerant deep-sea species. It opens up new opportunities for future studies of environmental adaptation and metabolic pathway utilization, for example, through incorporating environment-specific features like the altered fatty acid compositions in different temperatures and pressures (14) or the differential expression of key metabolic genes under different environmental conditions (31, 33, 36). The WP3 model also provides a framework for integrating additional parameters, such as enzyme thermostability (69) or context-specific information (70) during the study of temperature and pressure adaptations. The experimental accessibility of WP3 would make it possible to verify extensions to the model. Finally, future studies combining molecular evolution and metabolic simulation of the group 1 and group 2 *Shewanella* species could lead to a better understanding of bacterial adaptations to low-temperature and high-pressure environments and permit the exploration of metabolic potentials in the deep sea.

## MATERIALS AND METHODS

### Ortholog mapping and phylogenomic reconstruction of the *Shewanella* genus.

An updated phylogeny of the *Shewanella* genus was constructed based on conserved single-copy genes (CSCGs). A data set of 24 *Shewanella* genomes was downloaded from the KEGG database (71). Five additional genomes were used as the outgroup for rooting the *Shewanella* phylogeny, including *Pseudoalteromonas haloplanktis*, *Colwellia psychrerythraea*, *Psychromonas ingrahamii*, *Photobacterium profundum*, and *Moritella viscosa*. An initial ortholog mapping among these species was identified using a bidirectional best hit BLAST analysis as defined in a previous study (72). The ortholog mapping was further refined based on a consensus of additional evidence from other sources, including a published ortholog table of the *Shewanella* genus (25), the KEGG Orthology database (71), and automated predictions by OrthoMCL (73). From analyzing ortholog groups that were consistently defined by all of the above-mentioned approaches, CSCGs were identified as the orthologs that occurred once and only once in each of the genomes analyzed. Individual alignments were constructed on the protein sequences of each CSCG using MUSCLE version 3.8.31 (74). The alignments were then concatenated to create a master alignment of the CSCGs in *Shewanella* and the outgroups. RAxML version 8.2.3 (75) was used for reconstructing a maximum-likelihood protein phylogeny using the JTT substitution model with the GAMMA model of rate heterogeneity. Branch support values were estimated by performing bootstrapping with 100 replications.

### Development of the genome-scale metabolic reconstruction.

The WP3 metabolic reconstruction was developed using version 0.27 of the PSAMM software package (23). The reconstruction was represented in a YAML format that is designed to represent variable model definitions and simulation conditions. Simulations with the model were performed in PSAMM using the IBM ILOG CPLEX Optimizer version 12.6.2 linear programming solver. An initial reconstruction was first developed based on ortholog mapping to the existing metabolic reconstructions of *S. oneidensis* MR-1, *Shewanella* sp. MR-4, *S. denitrificans* strain OS217, and *Shewanella* sp. W3-18-1 (25). The orthologs were identified according to a global mapping of ortholog clusters among all *Shewanella* species (described in the paragraph above). Gene-protein-reaction (GPR) associations in the initial WP3 reconstruction were mapped from conserved genes in the modeled species, following logic expressions that represent the “AND” and “OR” relationships of enzyme-coding genes. The “AND” logic was used to indicate multiple subunits of an enzyme complex, and the “OR” logic was used to indicate alternative enzymes. A GPR association was introduced from existing reconstructions only if orthologs were identified in the WP3 genome for all subunits of at least one alternative enzyme. The WP3 reconstruction was further expanded through manual curation by referencing existing annotations in the KEGG (71), SEED (42), and BioCyc (76) databases. Additional considerations in the manual curation process included examining genomic context using the SEED viewer tool (77), searching for conserved sequence domains (78), and reviewing current literature (12, 14, 41, 79). Finally, metabolic gaps in the production of biomass components were identified using the PSAMM *gapfill* function (23). A number of gap-filling reactions were included to enable biomass production with experimentally confirmed carbon sources and electron acceptors (13). These gap reactions were further scrutinized through manual inspection of the biosynthetic pathways leading to the various biomass components and were reviewed with the *fluxcheck* function using the “--unrestricted” option in PSAMM to confirm their flux consistency. Stoichiometric consistency of the model was validated by using the *masscheck* function in PSAMM. Additional verification of the formula and charge balance was performed with the *formulacheck* and *chargecheck* functions. By default, the exchange reactions, compound sources or sinks (e.g., 4HBASink, 5DRIB_Sink, and AMOB_Sink), macromolecular synthesis equations (e.g., Core_Biomass, Growth, and PASYN_WP3_20C), and reactions involving the acyl carrier protein (ACP) or its apo form (e.g., ACPS1, ACPSc, and AGPEPHOS) were excluded from formula and charge checks due to the presence of undefined R or X groups in the metabolites.

### Formulating the biomass objective function.

A biomass equation was formulated in the WP3 reconstruction to simulate the production of components required for cell growth. The biomass equation incorporates the cellular composition of the total cellular carbohydrates, proteins, RNA, DNA, lipids, vitamins, and cofactors (see Table S1 in the supplemental material). Biomass compositions from experimental measurements of WP3 and evolutionarily related species were used as references for formulating the stoichiometry of the biomass equation. First, the composition of carbohydrates, proteins, DNA, RNA, and lipids was estimated using approximations from *S. oneidensis* MR-1 (24, 25). The addition of vitamins and cofactors into the WP3 biomass was achieved by using an approximation of the experimental measurements from *E. coli* as a representation of Gram-negative bacteria (80). Further calibration of the biomass composition in WP3 involved formulating the stoichiometry of the phosphatidic acid synthase reactions according to experimental measurements of branched-chain, unsaturated, and saturated fatty acids in this organism (14) (see Table S2). The overall biomass equation was scaled so that the stoichiometry of biomass components corresponds to their millimole (mmol) amounts in a gram of cell dry weight (gDW). This calibration enabled the comparison of computationally simulated biomass production levels with experimental measurements.

### Formulating the basal constraints of metabolic simulations.

A list of basal constraints was defined for exchange reactions in the model using the lower and upper bounds specified in Table S3 in the supplemental material. The basal constraints were used to set default bounds for the uptake of nutrient sources and the removal of metabolic by-products. For trace elements, vitamin precursors, and salts, the default bounds were unlimited in both directions, and for metabolic by-products, the lower bounds were set to zero while the upper bounds were unlimited, indicating that they can be freely released from the system. The basal constraints also defined the exchange reactions for 71 potential carbon sources and 13 electron acceptors in the model. The uptake of carbon sources and electron acceptors was blocked in the basal constraints and was defined during individual simulations. Unless otherwise specified (e.g., as defined in Table 1), the lower bound of the sole carbon source was set to −10 to limit its uptake to 10 mmol/liter, and the uptake of the sole electron acceptor was unlimited.

### Comparing WP3 metabolic simulations with experimental results and the MR-1 model.

The growth of WP3 when utilizing a variety of sole carbon sources was examined in aerobic batch cultures using 50 ml of LMO-812 minimal medium (see Text S1 in the supplemental material) supplemented with alternative sole carbon sources at different concentrations (2 mM, 5 mM, 10 mM, 20 mM, or 40 mM). Cultures were grown in triplicate at 20°C and were continuously shaken at 200 rpm. The growth curve of WP3 was determined using turbidity measurements at an optical density of 600 nm (OD_600_). The growth measurements at early stationary phase were converted to gDW/liter of biomass concentration using a previously determined correlation between OD_600_ and dry weight in *Shewanella* species (24). To simulate the experimental growth conditions, the PSAMM *fba* function was applied to perform FBA simulations using the biomass equation as the objective function. The exchange of carbon, nitrogen, sulfur, and phosphorus nutrients was constrained based on their availability in the experimental medium (Table 1), the exchange of oxygen was unlimited to simulate aerobic respiration with oxygen as the sole electron acceptor, and other exchange reactions were defined with the basal constraints. The unit of the uptake fluxes was assigned to mmol/liter, which corresponds to the unit of nutrient concentration in the experimental medium. Since the biomass equation in WP3 was calibrated to reflect the millimole (mmol) amounts of biomass components in a gram of cell dry weight (gDW), the biomass concentrations were predicted based on the biomass fluxes (*v*_*B*_* *gDW/liter).

Comparisons of the WP3 with the MR-1 model were performed by simulating the aerobic growth of the organisms using 28 sole carbon sources that have been experimentally confirmed to support the growth of either WP3 (13) or MR-1 (24, 25) (see Fig. S2 in the supplemental material). The latest metabolic reconstruction of MR-1, iMR1_799 (25) was used in all MR-1 simulations performed in this study. The simulations with both models were set up using the basal constraints with default bounds for the sole carbon sources (i.e., [−10, 1,000]) and the sole electron acceptor, oxygen (i.e., [−1,000, 1,000]). The biomass yields were calculated through dividing the biomass flux by the uptake fluxes of the carbon source and the electron acceptor.

### Metabolic simulation of mutant phenotypes.

Mutant strains of WP3 were simulated in the metabolic model by setting a flux limit of [0, 0] for all reactions catalyzed by the gene being knocked out. A list of enzymes involved in ATP production, PMF generation, and redox activities is provided in Table 2, along with their corresponding reactions, functional roles, and gene associations in the WP3 model. Medium conditions were set in the WP3 model using the basal constraints with uptake enabled for a sole carbon source (lactate or GlcNac) and a sole electron acceptor (O_2_ or fumarate). The carbon source was constrained to a maximum uptake of 10* *mmol/liter, and the electron acceptor was unlimited. For simulations with fumarate as the electron acceptor, the succinate/fumarate antiporter reaction, SUCFUMtdc, was blocked, as it has been noted to be able to form artificial loops with other transporters (80), and the fumarate hydrogen symporters FUMt4 and FUMt4_2 were also blocked to prevent utilization of fumarate as an additional carbon source. When GlcNac was used as the sole carbon source, the lactate dehydrogenase and glycerol-3-phosphate dehydrogenase reactions were blocked to prevent the formation of artificial loops in NADH cycling. Metabolic reaction fluxes were determined by optimizing the biomass objective function using *fba* with the *l1min* loop removal approach implemented in PSAMM (23, 81). Additional analysis of flux variability was performed on internal reactions with the *fva* function in PSAMM by fixing the biomass flux to its maximum. The reaction flux for Fdh was calculated based on the sum of fluxes through the FDH9 and FDH10 reactions, and the reaction flux for Ndh was calculated based on the sum of fluxes through the NADH4, NADH12, and NADH14 reactions. All other fluxes were obtained directly from the FBA and FVA simulations according to the reactions listed in Table 2.

**TABLE 2:** Metabolic enzymes involved in ATP production and PMF generation, with their corresponding reactions, functional roles, and gene associations in the WP3 model^a^

Enzyme	Reaction identifier(s)	Function	Gene association(s)
Pta	PTAr	Phosphotransacetylase	swp_1948
AckA	ACKr	Acetate kinase	swp_1949
ATPase	ATPS4r	ATP synthase	swp_5155 AND swp_5156 AND swp_5157 AND swp_5158 AND swp_5159 AND swp_5160 AND swp_5161
Pyk	PYK	Pyruvate kinase	swp_2388
Pfl	PFL	Formate *C*-acetyltransferase	swp_1952
Xpk	XPK	Xylulose-5-phosphate phosphoketolase	swp_3738
Ndh	NADH4, NADH12, NADH14	NADH dehydrogenase	swp_1298 OR swp_2117 OR swp_4014
Fdh	FDH9, FDH10	Formate dehydrogenase	(swp_5024 AND swp_5025 AND swp_5023) OR (swp_5027 AND swp_5028 AND swp_5029)

a A schematic of key reactions and comparisons of biomass and reaction fluxes is shown in Fig. 4.

### Metabolic simulations of the NAD^+^/NADH homeostasis.

The NAD^+^/NADH homeostasis was used as an approximation for investigating redox states in the WP3 model and the MR-1 model, iMR1_799 (25). To simulate the NAD^+^/NADH homeostasis, an artificial reaction, NAD^+^ + H^+^ ⇔ NADH (denoted EQ1), was introduced to the model to account for differences in the concentrations of NAD^+^ and NADH. First, a robustness analysis was performed by varying the flux value of EQ1 while optimizing the biomass production. This was performed using the *robustness* function in PSAMM (23), where flux values of EQ1 were probed in the range of [−10, 10] at 500 steps. For each step, FBA simulation was performed with the *l1min* loop removal, and the simulated ATPase flux was plotted with the corresponding flux of EQ1 (see Fig. S3 and S4 in the supplemental material). Next, a linear model was fit to the data using the equation *v*_ATPase_ = *k* · *v*_EQ1_ + *b*, where *v*_ATPase_ was the flux of the ATPase reaction and *v*_EQ1_ was the flux of the EQ1 reaction. To identify the connections between ATPase activity (i.e., ATP production or PMF generation) and the redox state of a cell, the intersection of the linear model with the EQ1 axis was used to determine the difference in NAD^+^ and NADH concentrations when the ATPase reaction flux approached zero. A negative intersection of the linear model on the EQ1 axis would indicate (NAD^+^) − (NADH) < 0, suggesting that the homeostasis was pushed toward generating more NADH; a positive intersection would indicate (NAD^+^) − (NADH) > 0, suggesting that the homeostasis was pushed toward generating more NAD^+^. Both the WP3 and MR-1 models were simulated using basal constraints, with the addition of fumarate as the anaerobic electron acceptor paired with one of 12 sole carbon sources that are growth supporting in both models (Fig. 5; see also Fig. S3 and S4). The exchange flux of the sole carbon source was constrained to [−10, 1,000], and the exchange of the electron acceptor was unlimited. The fumarate transport reactions SUCFUMtdc, FUMt4, and FUMt4_2 were blocked as mentioned above to avoid artificial loops and prevent the utilization of fumarate as an additional carbon source. The proton-pumping NADH dehydrogenase in MR-1 was blocked due to the lack of evidence of its participation in energy metabolism (8, 24, 25). All other internal reactions in the WP3 and MR-1 models were constrained based on the reaction reversibility using default settings in PSAMM.
