# Supplementary material for: A Genome-Scale Model of Shewanella piezotolerans Simulates Mechanisms of Metabolic Diversity and Energy Conservation
Source: mSystems. 2017 Mar 28;2(2):e00165-16. doi: 10.1128/mSystems.00165-16 (PMC5371395; doi:10.1128/mSystems.00165-16)
Supplement: TABLE S1 [file sys002172099st5.pdf]

Table S1. Stoichiometry of the biomass compounds involved in the biomass synthesis equation of the WP3 GEM.

Compound_ID	Stoichiometry	Compound_Name	Molecular Weight (g/mol)	Weight (g/gDW)
cpd_12dag3p	0.000078	1,2-Diacyl-sn-glycerol 3-phosphate	645.2608	5.033034E-05
cpd_12dgr	0.139174	1,2-Diacylglycerol	567.2988	7.895324E-02
cpd_5mthf	0.050000	5-Methyltetrahydrofolate	458.4620	2.292310E-02
cpd_accoa	0.000050	Acetyl-CoA	805.5480	4.027740E-05
cpd_amp	0.001000	AMP	345.2160	3.452160E-04
cpd_coa	0.000006	CoenzymeA	763.5120	4.581072E-06
cpd_dna	0.001819	DNA	30792.5160	5.601159E-02
cpd_fad	0.000050	FAD	783.5480	3.917740E-05
cpd_lps_Core	0.011270	LPS	3052.1980	3.439675E-02
cpd_nad	0.002150	NAD	662.4280	1.424220E-03
cpd_nadh	0.000050	NADH	663.4360	3.317180E-05
cpd_nadp	0.000130	NADP	740.3900	9.625070E-05
cpd_nadph	0.000400	NADPH	741.3980	2.965592E-04
cpd_pe	0.078517	Phosphatidylethanolamine	690.3468	5.420396E-02
cpd_peptx	0.031434	Peptidoglycan subunit crosslinked	899.8900	2.828714E-02
cpd_pgly	0.022716	Phosphatidylglycerol	720.3468	1.636340E-02
cpd_protein	0.000551	Protein	1083383.9340	5.969445E-01
cpd_rna	0.000003	RNA	32013.8140	9.604144E-05
cpd_succoa	0.000003	Succinyl-CoA	862.5760	2.587728E-06
cpd_udpg	0.003000	UDP-Glucose	564.2860	1.692858E-03
cpd_glycogen	0.472431	Glycogen	162.1400	7.659996E-02
cpd_ptrc	0.035000	Putrescine	90.1720	3.156020E-03
cpd_spmd	0.007000	Spermidine	148.2760	1.037932E-03
cpd_k	0.169185	K+	38.9637	6.592084E-03
cpd_nh4	0.011279	NH4	18.0390	2.034622E-04
cpd_mg2	0.007519	Mg2+	23.9850	1.803515E-04
cpd_ca2	0.004512	Ca2+	39.9626	1.802956E-04
cpd_fe2	0.006767	Fe2+	55.9349	3.785345E-04
cpd_cu2	0.012698	Cu2+	63.5460	8.069333E-04
cpd_mn2	0.012698	Mn2+	54.9380	6.976254E-04
cpd_mobd	0.012698	Molybdate	159.9400	2.030984E-03
cpd_cobalt2	0.012698	Co2+	58.9332	7.483581E-04
cpd_cl	0.019048	Chloride	34.9689	6.660743E-04
cpd_so4	0.015873	SO4	96.0620	1.524794E-03
cpd_pydx5p	0.000223	Pyridoxal 5'-phosphate	245.1270	5.474503E-05
cpd_pheme	0.000223	Protoheme	690.6269	1.542400E-04
cpd_udcpdp	0.000055	Undecaprenyl diphosphate	924.2580	5.116008E-05
cpd_chor	0.000223	Chorismate	224.1680	5.006419E-05
cpd_amet	0.000223	S-Adenosyl-L-methionine	399.4520	8.921095E-05
cpd_ribflv	0.000223	Riboflavin	376.3690	8.405574E-05
cpd_sheme	0.000223	Siroheme	908.6969	2.026394E-04
cpd_ubq8h2	0.000223	Ubiquinol-8	729.1430	1.625989E-04
cpd_mqn7	0.000223	Menaquinone 7	719.1510	1.603707E-04
cpd_cobamcoa	0.000223	Cobamide coenzyme	1579.5818	3.522467E-04
cpd_thmpp	0.000223	Thiamine diphosphate	422.2950	9.417179E-05
cpd_btn	0.000002	Biotin	244.3100	4.886200E-07
