# Supplementary material for: A Genome-Scale Model of Shewanella piezotolerans Simulates Mechanisms of Metabolic Diversity and Energy Conservation
Source: mSystems. 2017 Mar 28;2(2):e00165-16. doi: 10.1128/mSystems.00165-16 (PMC5371395; doi:10.1128/mSystems.00165-16)
Supplement: TABLE S2 [file sys002172099st6.pdf]

Table S2. Stoichiometry of the fatty acid components in the lipid biosynthesis equation of the WP3 GEM. The stoichiometry of unsaturated, saturated, and branch-chain fatty acids were calibrated based on experimental measurements of the WP3 fatty acid composition at 20°C and 0.1 MPa (Wang et al., 2009).

Compound_ID	Type	Stoichiometry
fa11ACP	Iso-C17:0	0.2210
fa13ACP	Iso-C13:0	0.0649
fa1ACP	Iso-C14:0	0.2040
fa3ACP	iso-C15:0	0.0759
fa6ACP	Iso-C16:0	0.2550
hdeACP	n-C16:1	0.1346
hpdACP	n-C17:0	0.0510
hpdeACP	C17:1	0.4250
ocdACP	n-C18:0	0.0340
octeACP	n-C18:1	0.0434
palmACP	n-C16:0A	0.2210
pdACP	n-C15:0	0.0340
pdeACP	C15:1	0.2040
epa	20:5	0.0321
