# Supplementary material for: A Genome-Scale Model of Shewanella piezotolerans Simulates Mechanisms of Metabolic Diversity and Energy Conservation
Source: mSystems. 2017 Mar 28;2(2):e00165-16. doi: 10.1128/mSystems.00165-16 (PMC5371395; doi:10.1128/mSystems.00165-16)
Supplement: TABLE S3 [file sys002172099st7.pdf]

Table S3. Basal constraints for metabolic simulations performed in the WP3 and MR-1 models (Materials and Methods). Compound ID/Name: the identifiers/names of extracellular compounds with defined exchange reactions, which were used to simulate the availability of nutrients and the removal of metabolic byproducts. The compound identifiers were shown for both the WP3 and MR-1 models. Lower/Upper Bound: basal constraints for the lower and upper bounds of exchange reaction fluxes. Negative lower bounds would indicate compounds provided as nutrient sources to the model, and a lower bound of zero would indicate a compound that could only be released as a metabolic byproduct but not acquired from the environment. Type: classification of the exchange compounds. Growth supporting in WP3: the growth supporting carbon sources and terminal electron acceptors were marked as TRUE in this column.

WP3 Compound ID	MR-1 Compound ID	Compound Name	Lower Bound	Upper Bound	Type	Growth Supporting in WP3
cpd_h[e]	h[e]	H+	-1000	1000	Basal media	-
cpd_h2o[e]	h2o[e]	H2O	-1000	1000	Basal media	-
cpd_na1[e]	na1[e]	Sodium	-1000	1000	Basal salt media	-
cpd_ca2[e]	ca2[e]	Calcium	-1000	1000	Basal salt media	-
cpd_cl[e]	cl[e]	Chloride	-1000	1000	Basal salt media	-
cpd_k[e]	k[e]	K+	-1000	1000	Basal salt media	-
cpd_mg2[e]	mg2[e]	Mg	-1000	1000	Basal salt media	-
cpd_nh4[e]	nh4[e]	Ammonium	-1000	1000	Basal salt media	-
cpd_pi[e]	pi[e]	Phosphate	-1000	1000	Basal salt media	-
cpd_so4[e]	so4[e]	Sulfate	-1000	1000	Basal salt media	-
cpd_cobalt2[e]	cobalt2[e]	Co2+	-1000	1000	Trace element	-
cpd_cu2[e]	cu2[e]	Cu2+	-1000	1000	Trace element	-
cpd_fe2[e]	fe2[e]	Fe2+	-1000	1000	Trace element	-
cpd_mn2[e]	mn2[e]	Mn2+	-1000	1000	Trace element	-
cpd_mobd[e]	mobd[e]	Molybdate	-1000	1000	Trace element	-
cpd_pmcoa[e]	pmcoa[e]	Pimeloyl-CoA	-1000	1000	Vitamin precursor	-
cpd_cbl1[e]	cbl1[e]	Cob(I)alamin	-1000	1000	Vitamin solution	-
cpd_ac[e]	ac[e]	Acetate	0	1000	Carbon source	TRUE
cpd_acgam[e]	acgam[e]	N-Acetyl-D-glucosamine	0	1000	Carbon source	TRUE
cpd_adn[e]	adn[e]	Adenosine	0	1000	Carbon source	TRUE
cpd_ala-D[e]	ala-d[e]	D-Alanine	0	1000	Carbon source	TRUE
cpd_ala-L[e]	ala-l[e]	L-Alanine	0	1000	Carbon source	TRUE
cpd_asp-L[e]	asp-l[e]	L-Aspartate	0	1000	Carbon source	TRUE
cpd_bgl[e]	bgl[e]	Cellobiose	0	1000	Carbon source	TRUE
cpd_chitin[e]	chitin[e]	Chitin	0	1000	Carbon source	TRUE
cpd_cytd[e]	cytd[e]	Cytidine	0	1000	Carbon source	TRUE
cpd_dad-2[e]	dad-2[e]	Deoxyadenosine	0	1000	Carbon source	TRUE
cpd_damp[e]	damp[e]	dAMP	0	1000	Carbon source	TRUE
cpd_dcmp[e]	dcmp[e]	dCMP	0	1000	Carbon source	TRUE
cpd_dcyt[e]	dcyt[e]	Deoxycytidine	0	1000	Carbon source	TRUE
cpd_dna[e]	dna[e]	DNA	0	1000	Carbon source	TRUE
cpd_dodcan[e]	dodca[e]	Dodecanoic acid	0	1000	Carbon source	TRUE
cpd_duri[e]	duri[e]	Deoxyuridine	0	1000	Carbon source	TRUE
cpd_for[e]	for[e]	Formate	0	1000	Carbon source	TRUE
cpd_fum[e]	fum[e]	Fumarate	0	1000	Carbon source	TRUE
cpd_gal[e]	gal[e]	D-Galactose	0	1000	Carbon source	TRUE
cpd_galactan[e]	galactan[e]	Galactan	0	1000	Carbon Source	TRUE
cpd_glc-D[e]	glc-d[e]	D-Glucose	0	1000	Carbon source	TRUE
cpd_glu-L[e]	glu-l[e]	L-Glutamate	0	1000	Carbon source	TRUE
cpd_gly-asp-L[e]	gly-asp-l[e]	Glycyl-L-aspartic acid	0	1000	Carbon source	TRUE
cpd_gly-glu-L[e]	gly-glu-l[e]	Glycyl-L-glutamic acid	0	1000	Carbon source	TRUE
cpd_gly[e]	gly[e]	Glycine	0	1000	Carbon source	TRUE
cpd_glyc-R[e]	glyc-r[e]	(R)-Glycerate	0	1000	Carbon source	TRUE
cpd_glyc[e]	glyc[e]	Glycerol	0	1000	Carbon source	TRUE
cpd_hdcan[e]	hdca[e]	hexadecanoate (n-C16:0)	0	1000	Carbon source	TRUE
cpd_ile-L[e]	ile-l[e]	L-Isoleucine	0	1000	Carbon source	TRUE
cpd_lac-D[e]	lac-d[e]	D-Lactate	0	1000	Carbon source	TRUE
cpd_lac-L[e]	lac-l[e]	L-Lactate	0	1000	Carbon source	TRUE
cpd_lami[e]	lami[e]	laminarin	0	1000	Carbon source	TRUE
cpd_leu-L[e]	leu-l[e]	L-Leucine	0	1000	Carbon source	TRUE
cpd_mal-L[e]	mal-l[e]	L-Malate	0	1000	Carbon source	TRUE
cpd_malt[e]	malt[e]	Maltose	0	1000	Carbon source	TRUE
cpd_malthp[e]	malthp[e]	Maltoheptaose	0	1000	Carbon source	TRUE
cpd_malthx[e]	malthx[e]	Maltohexaose	0	1000	Carbon source	TRUE
cpd_maltpt[e]	maltpt[e]	Maltopentaose	0	1000	Carbon source	TRUE
cpd_malttr[e]	malttr[e]	Maltotriose	0	1000	Carbon source	TRUE
cpd_maltttr[e]	maltttr[e]	Maltotetraose	0	1000	Carbon source	TRUE

cpd_ocdcan[e]	ocdca[e]	Octadecanoate (n-C18:0)	0	1000	Carbon source	TRUE
cpd_panose[e]	panose[e]	Panose	0	1000	Carbon Source	TRUE
cpd_ppa[e]	ppa[e]	Propionate	0	1000	Carbon source	TRUE
cpd_pro-L[e]	pro-l[e]	L-Proline	0	1000	Carbon source	TRUE
cpd_ptrc[e]	ptrc[e]	Putrescine	0	1000	Carbon source	TRUE
cpd_pyr[e]	pyr[e]	Pyruvate	0	1000	Carbon source	TRUE
cpd_ser-L[e]	ser-l[e]	L-Serine	0	1000	Carbon source	TRUE
cpd_succ[e]	succ[e]	Succinate	0	1000	Carbon source	TRUE
cpd_thr-L[e]	thr-l[e]	L-Threonine	0	1000	Carbon source	TRUE
cpd_ttdcan[e]	ttdca[e]	tetradecanoate (C14:0)	0	1000	Carbon source	TRUE
cpd_tyr-L[e]	tyr-l[e]	L-Tyrosine	0	1000	Carbon source	TRUE
cpd_uri[e]	uri[e]	Uridine	0	1000	Carbon source	TRUE
cpd_val-L[e]	val-l[e]	L-Valine	0	1000	Carbon source	TRUE
cpd_akg[e]	akg[e]	2-Oxoglutarate	0	1000	Carbon source	FALSE
cpd_asn-L[e]	asn-l[e]	L-Asparagine	0	1000	Carbon source	FALSE
cpd_dgmp[e]	dgmp[e]	dGMP	0	1000	Carbon source	FALSE
cpd_dgsn[e]	dgsn[e]	Deoxyguanosine	0	1000	Carbon source	FALSE
cpd_dtmp[e]	dtmp[e]	dTMP	0	1000	Carbon source	FALSE
cpd_etoh[e]	etoh[e]	Ethanol	0	1000	Carbon source	FALSE
cpd_gln-L[e]	gln-l[e]	L-Glutamine	0	1000	Carbon source	FALSE
cpd_glyclt[e]	glyclt[e]	Glycolate	0	1000	Carbon source	FALSE
cpd_hxan[e]	hxan[e]	Hypoxanthine	0	1000	Carbon source	FALSE
cpd_indole[e]	indole[e]	Indole	0	1000	Carbon source	FALSE
cpd_ins[e]	ins[e]	Inosine	0	1000	Carbon source	FALSE
cpd_lys-L[e]	lys-l[e]	L-Lysine	0	1000	Carbon source	FALSE
cpd_met-L[e]	met-l[e]	L-Methionine	0	1000	Carbon source	FALSE
cpd_thym[e]	thym[e]	Thymine	0	1000	Carbon source	FALSE
cpd_thymd[e]	thymd[e]	Thymidine	0	1000	Carbon source	FALSE
cpd_trp-L[e]	trp-l[e]	L-Tryptophan	0	1000	Carbon source	FALSE
cpd_ura[e]	ura[e]	Uracil	0	1000	Carbon source	FALSE
cpd_xan[e]	xan[e]	Xanthine	0	1000	Carbon source	FALSE
cpd_cro4[e]	cro4[e]	chromate	0	1000	Electron acceptor	TRUE
cpd_dmso[e]	dmso[e]	Dimethyl sulfoxide	0	1000	Electron acceptor	TRUE
cpd_fe3[e]	fe3[e]	Fe3+	0	1000	Electron acceptor	TRUE
cpd_h2o2[e]	h2o2[e]	Hydrogen peroxide	0	1000	Electron acceptor	TRUE
cpd_mn4o[e]	mn4o[e]	Manganese(IV) oxide	0	1000	Electron acceptor	TRUE
cpd_no2[e]	no2[e]	Nitrite	0	1000	Electron acceptor	TRUE
cpd_no3[e]	no3[e]	Nitrate	0	1000	Electron acceptor	TRUE
cpd_o2[e]	o2[e]	O2	0	1000	Electron acceptor	TRUE
cpd_tmao[e]	tmao[e]	Trimethylamine N-oxide	0	1000	Electron acceptor	TRUE
cpd_tsul[e]	tsul[e]	Thiosulfate	0	1000	Electron acceptor	TRUE
cpd_tttnt[e]	tttnt[e]	tetrathionate	0	1000	Electron acceptor	TRUE
cpd_urnyl[e]	urnyl[e]	Uranyl	0	1000	Electron acceptor	TRUE
cpd_cobalt3[e]	cobalt3[e]	Co3+	0	1000	Electron acceptor	FALSE
cpd_co2[e]	co2[e]	CO2	0	1000	Metabolic byproduct	-
cpd_CrOH3[e]	CrOH3[e]	Cr(OH)3	0	1000	Metabolic byproduct	-
cpd_dms[e]	dms[e]	Dimethyl sulfide	0	1000	Metabolic byproduct	-
cpd_h2s[e]	h2s[e]	Hydrogen sulfide	0	1000	Metabolic byproduct	-
cpd_so3[e]	so3[e]	Sulfite	0	1000	Metabolic byproduct	-
cpd_tma[e]	tma[e]	Trimethylamine	0	1000	Metabolic byproduct	-
cpd_urdio[e]	urdio[e]	Uranium dioxide	0	1000	Metabolic byproduct	-
cpd_urea[e]	urea[e]	Urea	0	1000	Metabolic byproduct	-
