# Supplementary figures and images for: A Genome-Scale Model of Shewanella piezotolerans Simulates Mechanisms of Metabolic Diversity and Energy Conservation
Source: mSystems. 2017 Mar 28;2(2):e00165-16. doi: 10.1128/mSystems.00165-16 (PMC5371395; doi:10.1128/mSystems.00165-16)

A) ArgE, Group I

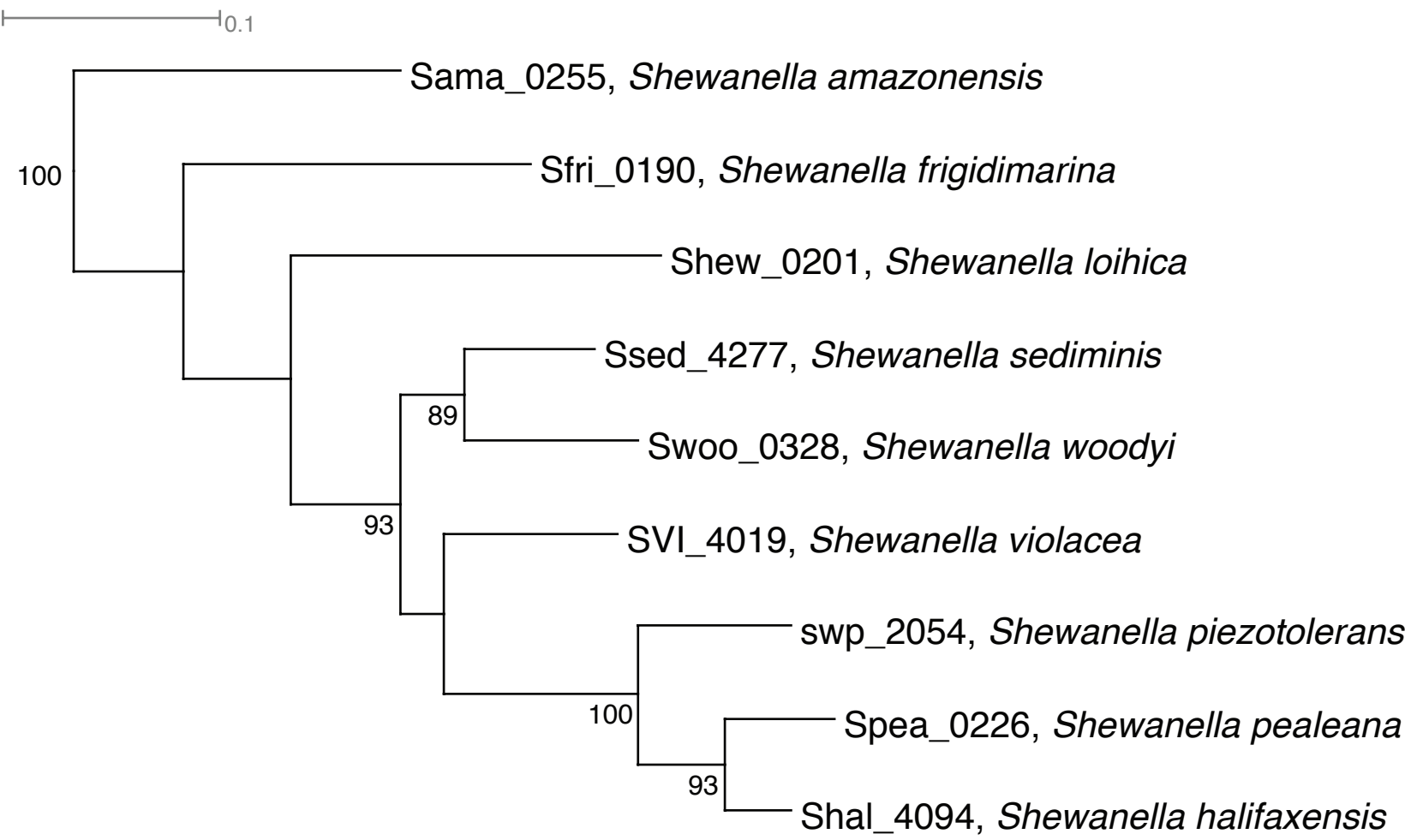

C) NagB, Group I

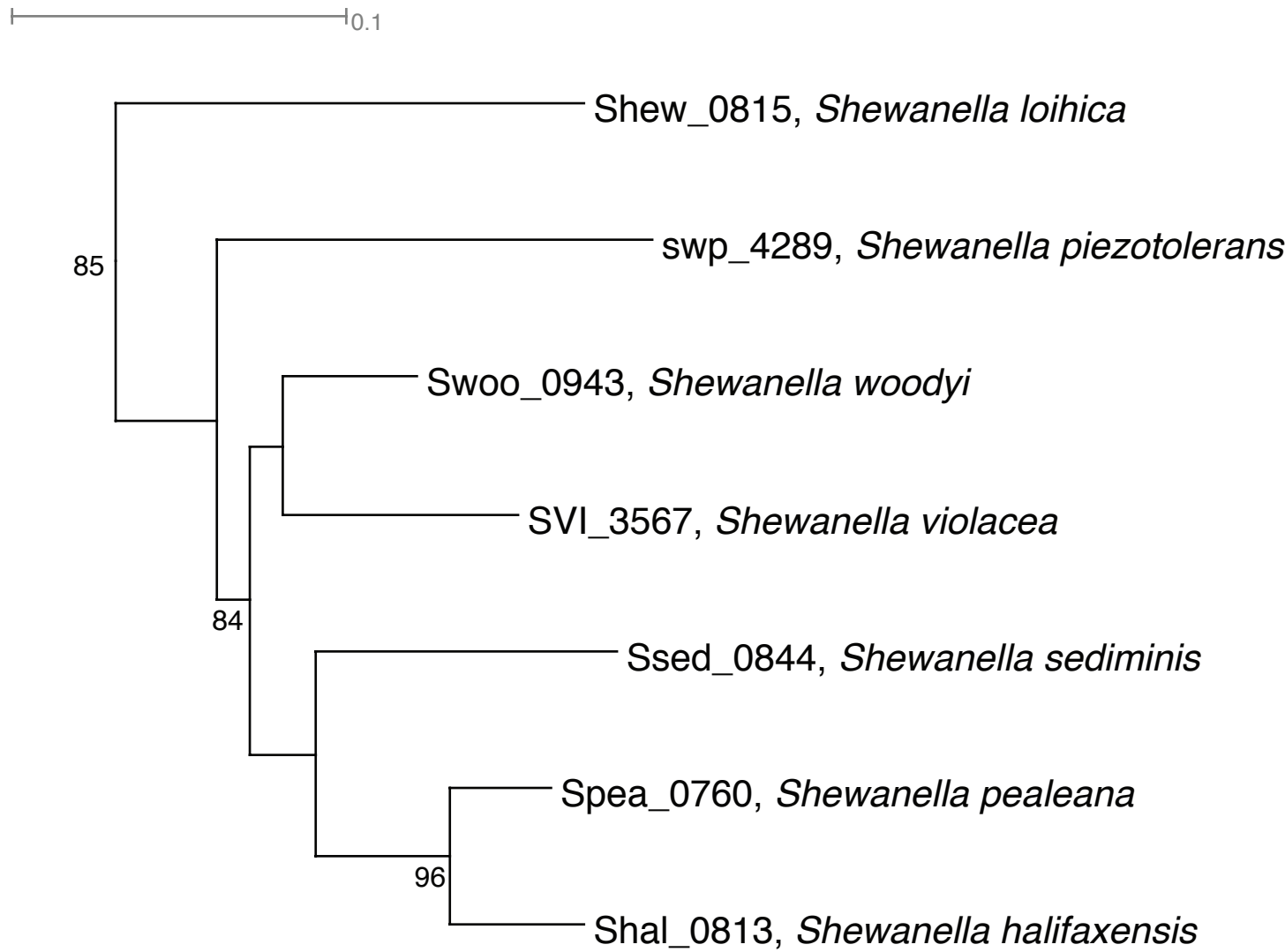

B) ArgE, Group II

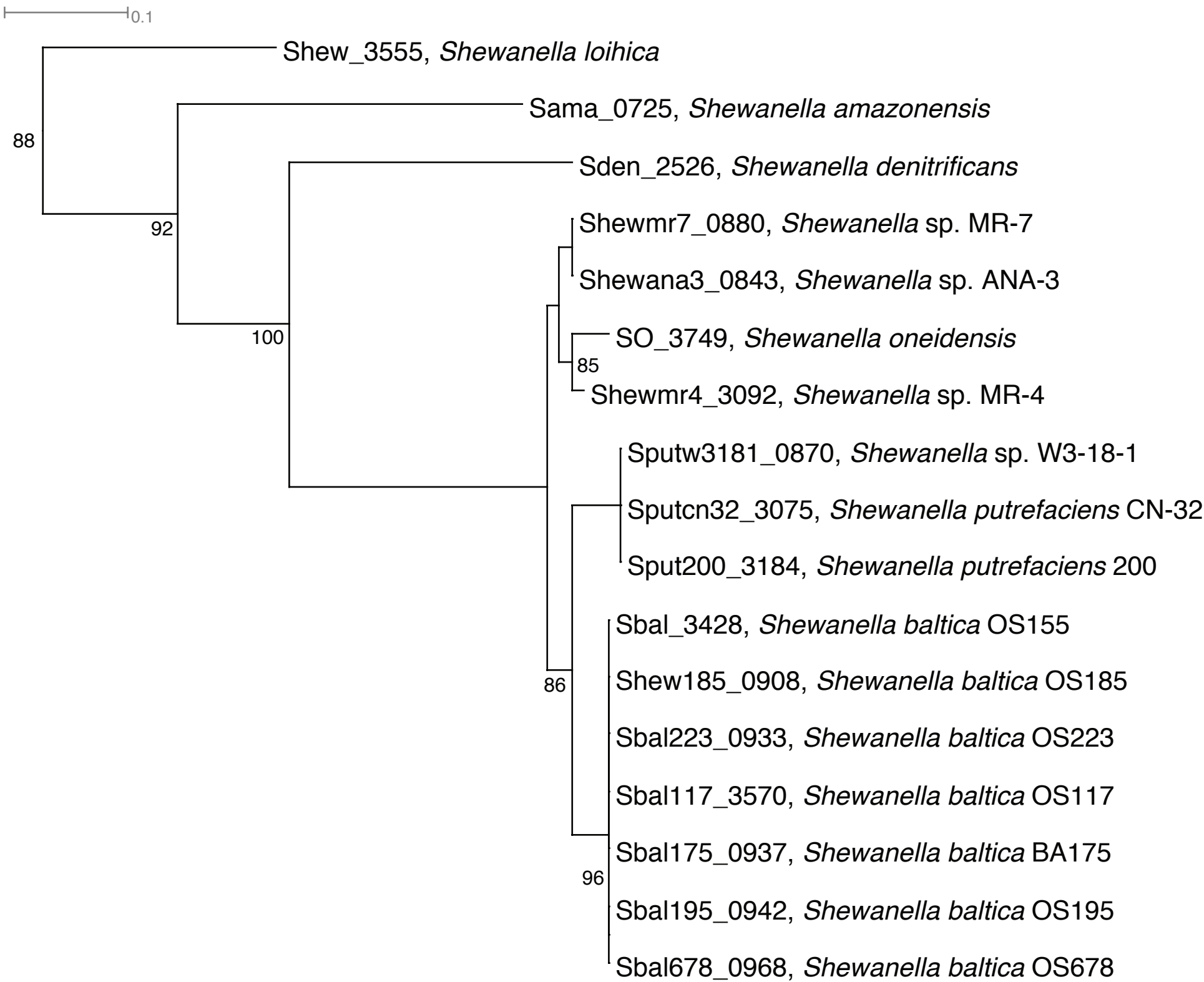

D) NagB, Group II

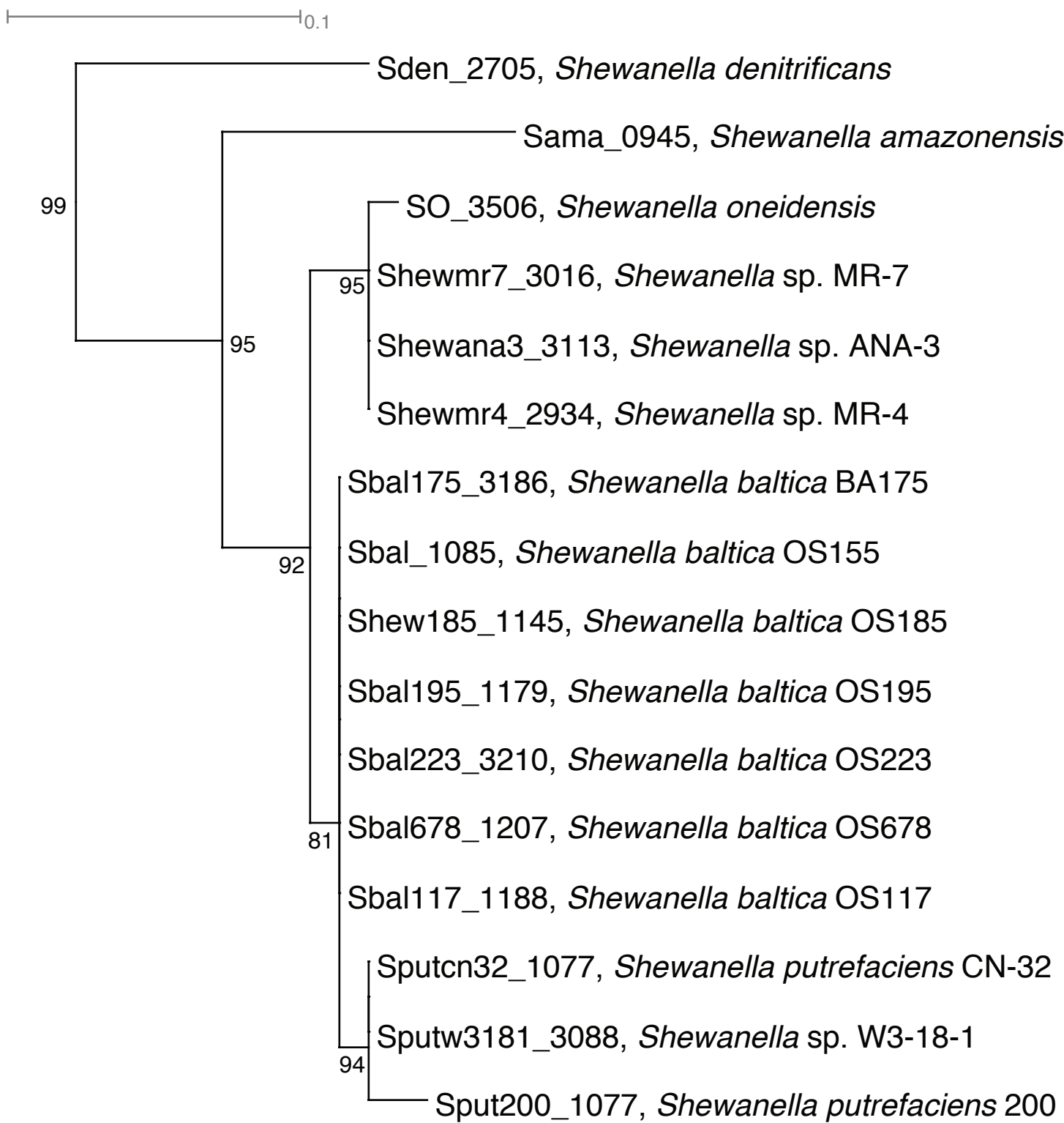

Supplement: FIG S1 [file sys002172099sf1.pdf]

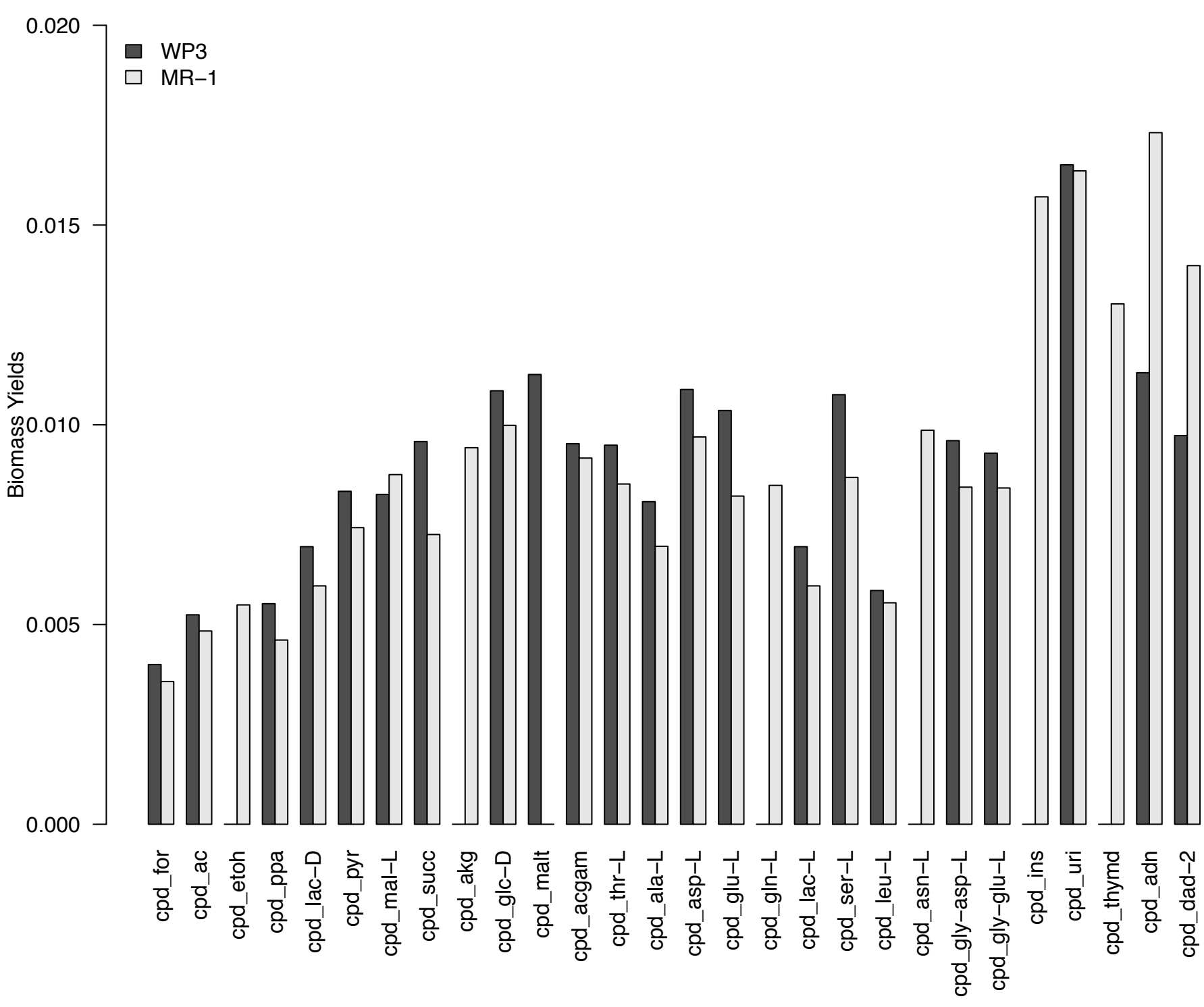

Supplement: FIG S2 [file sys002172099sf2.pdf]

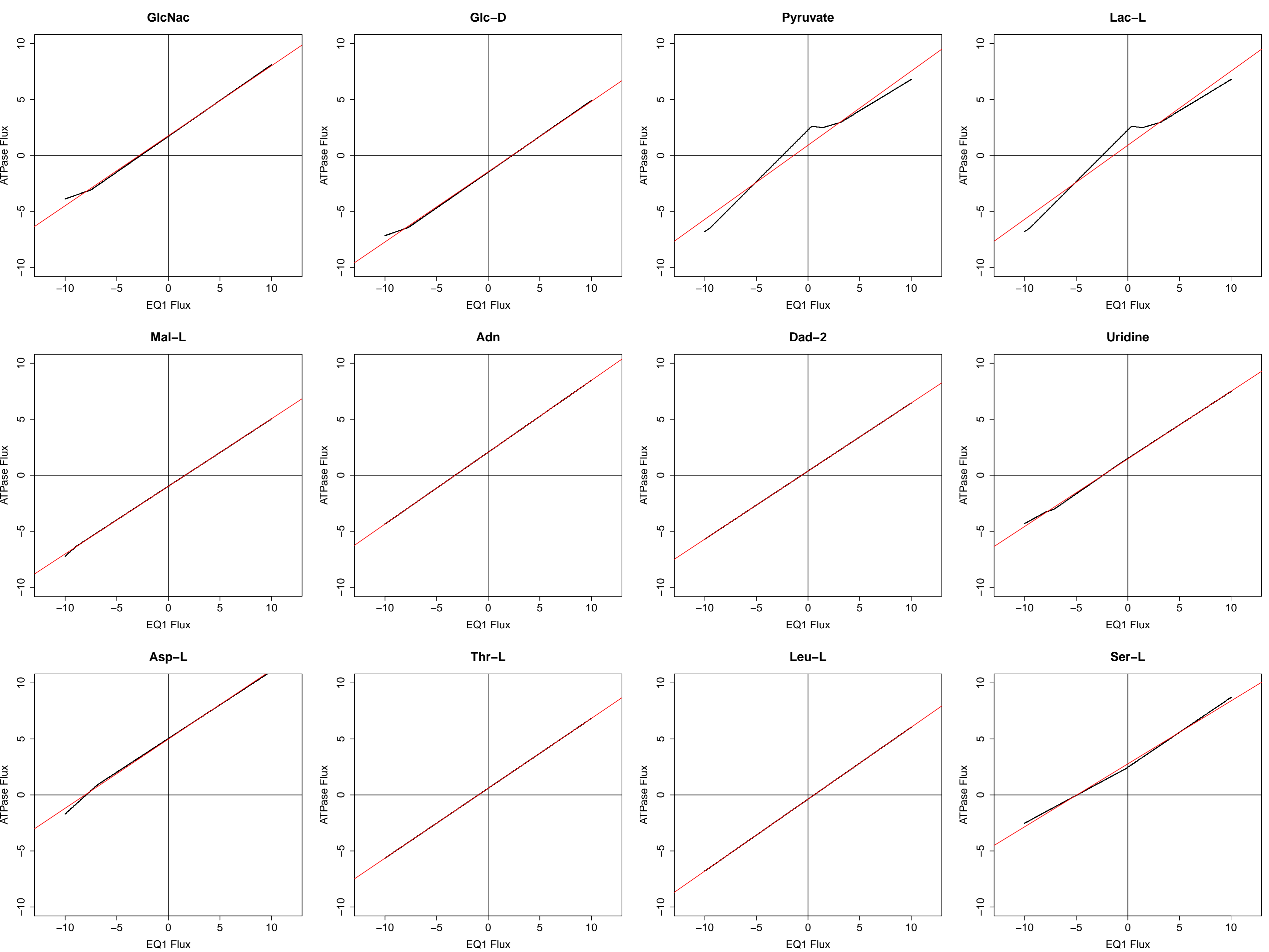

Supplement: FIG S3 [file sys002172099sf3.pdf]

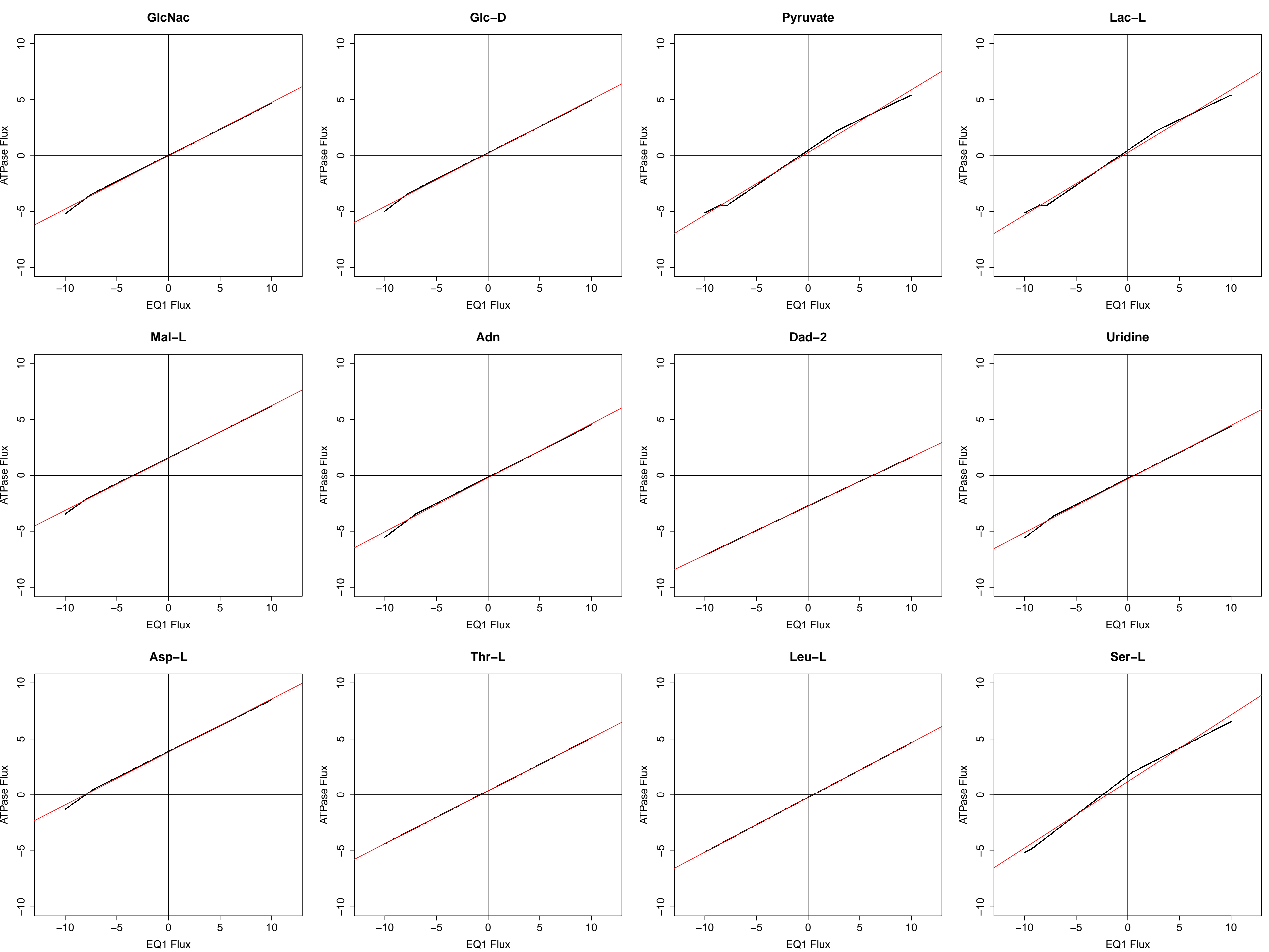

Supplement: FIG S4 [file sys002172099sf4.pdf]
