# Supplementary material for: A Genome-Scale Model of Shewanella piezotolerans Simulates Mechanisms of Metabolic Diversity and Energy Conservation
Source: mSystems. 2017 Mar 28;2(2):e00165-16. doi: 10.1128/mSystems.00165-16 (PMC5371395; doi:10.1128/mSystems.00165-16)
Supplement: TEXT S1 [file sys002172099s9.pdf]

Supplemental Text S1: Components of the LMO-812 minimal media used for the experimental culture of *Shewanella piezotolerans* WP3. Media components were adapted from a previously described defined marine media (1).

LMO-812: Full Media
(Adjust final pH to 7.0 with Na_2CO_3 solution)

Component	Amount
Full Marine Salt Solution	950 mL
Trace Element Mixture	1 mL
Vitamin Mixture	1 mL
Thiamine Solution	1 mL
Vitamin B12 Solution	1 mL
$NaHCO_3$ Solution	20 mL

Full Marine Salt Solution

Component	Amount
Distilled Water	950 mL
NaCl	26.0 g
$MgCl_2 \cdot 6\ H_2O$	5 g
$CaCl_2 \cdot 2\ H_2O$	1.4 g
Na_2SO_4	4.0 g
NH_4Cl	0.3 g
KH_2PO_4	0.1 g
KCl	0.5 g

Trace Element Mixture
(Adjust final pH of solution to 6.0 with NaOH solution)

Component	Amount
Distilled Water	1000 mL
EDTA	5.2 g
H_3BO_3	10 mg
$MnCl_2 \cdot 4\ H_2O$	5 mg
$FeSO_4 \cdot 7\ H_2O$	2100 mg
$CoCl_2 \cdot 6\ H_2O$	190 mg
$NiCl_2 \cdot 6\ H_2O$	24 mg
$CuCl_2 \cdot 2\ H_2O$	10 mg
$ZnSO_4 \cdot 7\ H_2O$	144 mg
$Na_2MoO_4 \cdot 2\ H_2O$	36 mg

Vitamin Mixture

Component	Amount
Sodium Phosphate, 10 mM, pH 7.1	100 mL
4-Aminobenzoic acid	4 mg
D(+)-Biotin	1 mg

Nicotinic Acid	10 mg
D(+)-Pantothenic Acid, Calcium Salt	5 mg
Pyridoxine dihydrochloride	15 mg

Thiamine Solution

**Component**	**Amount**
Sodium Phosphate, 10 mM, pH 3.4	100 mL
Thiamine chloride dihydrochloride	10 mg

Vitamin B12 Solution

**Component**	**Amount**
Distilled Water	100 mL
Cyanocobalamin	5 mg

Bicarbonate Solution

**Component**	**Amount**
Distilled Water	1000 mL
$NaHCO_3$	84 g

1. Widdel, Friedrich. 2005. 8.1.1. Media for sulphate-reducing bacteria, p 102-104. In HERMES, Handbook of Methods for Microbial Ecology, Sept 2005 Edition.
