# Supplementary material for: A Genome-Scale Model of Shewanella piezotolerans Simulates Mechanisms of Metabolic Diversity and Energy Conservation
Source: mSystems. 2017 Mar 28;2(2):e00165-16. doi: 10.1128/mSystems.00165-16 (PMC5371395; doi:10.1128/mSystems.00165-16)
Supplement: TABLE S4 [file sys002172099st8.pdf]

**Table S4.** Maximum and minimum flux values obtained from flux variability analysis (FVA) corresponding to the simulation condition in Figure 4D. FVA was performed in the WP3 wild type model and the ∆pta, ∆ackA, and ∆pta∆ackA mutant models with biomass production set to its maximum (Materials and Methods). Numbers in this table indicated raw values of the minimum and maximum fluxes before they were normalized by the biomass flux.

| Function | WT | | *∆pta* | | *∆ackA* | | *∆pta∆ackA* | |
|---|---|---|---|---|---|---|---|---|
| | Min | Max | Min | Max | Min | Max | Min | Max |
| **ATPase** | 1.72 | 1.72 | 0.46 | 0.59 | 0.20 | 0.20 | 0.20 | 0.20 |
| **Fdh** | 10.05 | 10.05 | 6.30 | 6.49 | 2.66 | 18.06 | 2.66 | 18.06 |
| **Ndh** | 7.11 | 7.11 | 9.92 | 9.92 | 2.53 | 75.85 | 2.53 | 75.85 |
| **Pta** | 6.35 | 6.35 | 0.00 | 0.00 | 0.00 | 0.00 | 0.00 | 0.00 |
| **AckA** | 12.45 | 12.45 | 12.97 | 13.03 | 0.00 | 0.00 | 0.00 | 0.00 |
| **Pyk** | 6.26 | 6.26 | 6.18 | 6.31 | 3.29 | 8.42 | 3.29 | 8.42 |
| **Xpk** | 6.09 | 6.09 | 12.97 | 13.03 | 0.00 | 0.00 | 0.00 | 0.00 |
| **Biomass** | 0.54 | 0.54 | 0.52 | 0.52 | 0.23 | 0.23 | 0.23 | 0.23 |
